# Intermittent Fasting and Healthy Aging in Older Adults: A Systematic Review of Cardiometabolic, Mental Health and Cognitive Outcomes with a Network Meta-Analysis of Anthropometric Measures

**DOI:** 10.3390/nu18091450

**Published:** 2026-04-30

**Authors:** Sergio Couto-Alfonso, María Carmen Cenit, Cristina María Sanz-Pérez, Isabel Iguacel

**Affiliations:** 1 Faculty of Health Sciences, University of Zaragoza, 50009 Zaragoza, Spain; scouto@unizar.es; 2 Department of Nutrition and Sustainable Animal Production, Estación Experimental del Zaidín (EEZ-CSIC), 18008 Granada, Spain; 3 Aragon Health Service, 50017 Zaragoza, Spain; 4 AgriFood Institute of Aragon (IA2), 50013 Zaragoza, Spain; 5 Biomedical Research Networking Center for the Physiopathology of Obesity and Nutrition (CIBEROBN), 28029 Madrid, Spain

**Keywords:** intermittent fasting, aging, metabolic health, cognition, mental health

## Abstract

**Background/Objective**: Intermittent fasting (IF) shows promise for metabolic and mental health benefits, but evidence in older adults remains limited. This study systematically evaluated the safety and effectiveness of IF in adults aged ≥60 years, comparing different protocols using network meta-analysis. **Methods**: Systematic review and network meta-analysis following Cochrane and PRISMA guidelines were conducted, producing a literature search until June 2025 across PubMed, Scopus, and ScienceDirect databases, with inclusion criteria comprising randomized controlled trials, clinical trials, and observational studies evaluating IF in adults ≥60 years. Network meta-analysis compared time-restricted eating (TRE), IF 5:2 method, Islamic Sunnah fasting (ISF), Healthy Living Diet and usual diet. The NMA was conducted exclusively using randomized controlled trials (RCTs; n = 7); pre–post trials and observational studies were included solely in the narrative systematic review component and did not contribute to any pooled NMA estimates. Observational data contributed exclusively to the narrative synthesis. **Results**: Thirty-one studies were included; seven RCTs were eligible for network meta-analysis. ISF and TRE 16:8 were most effective for weight (ISF: −2.36 kg; TRE 16:8: −1.92 kg) and BMI reduction (−0.81 and −1.01 kg/m^2^) without lean mass loss. Findings on cardiometabolic parameters, mental health, and cognitive function are based on the narrative synthesis of individual studies. Long-term structured IF was associated with improvements in standardized cognitive performance assessed via validated instruments. However, very restrictive eating windows (≤10 h) and prolonged fasting (>12.38 h) were associated with adverse outcomes, including lower cognitive scores and 58% increased cardiovascular mortality. **Conclusions**: TRE 16:8 and ISF showed the strongest comparative evidence for weight reduction in the RCT-based NMA, with acceptable short-term safety profiles in the included trials. In the narrative review, these protocols were associated with clinically meaningful improvements in body weight, metabolic markers, and blood pressure while generally preserving lean muscle mass in older adults. The cardiovascular mortality risk associated with very restrictive eating windows may emphasize the importance of moderate fasting approaches in this vulnerable population. Further long-term research is needed to confirm optimal protocols and identify at-risk subgroups.

## 1. Introduction

Intermittent fasting (IF) is a temporal dietary pattern alternating periods of restricted caloric intake with unrestricted feeding [1]. Unlike continuous energy restriction, IF emphasizes timing and pattern of food intake rather than total energy or nutrients, representing an alternative approach for weight loss and metabolic health promotion [2,3].

The most studied IF protocols include time-restricted eating (TRE), which restricts intake to an 8–12 h daily window aligned with circadian rhythms [4]; alternate-day fasting (ADF), alternating between fasting and normal eating days [5]; and the 5:2 method, involving five normal eating days followed by two days of severe caloric restriction [6]. Each regimen differs in fasting duration, frequency, and circadian alignment, potentially leading to heterogeneous health effects.

Proposed mechanisms underlying IF benefits include metabolic switching—where glycogen depletion shifts energy metabolism from glucose to ketone bodies, enhancing insulin sensitivity and metabolic flexibility—and autophagy promotion, a cellular recycling process contributing to stress resistance, neuroprotection, and reduced inflammation [7,8]. These adaptations may favorably impact cardiometabolic and neurological health, suggesting IF as a non-pharmacological strategy for disease prevention and healthy aging [9,10]. TRE may provide additional benefits through circadian alignment, optimizing energy utilization and hormonal regulation [4]. IF may also influence gut microbiota, potentially counteracting age-related dysbiosis that contributes to chronic inflammation and frailty.

The aging population is expanding rapidly, with a high prevalence of cardiovascular disease, type 2 diabetes, and neurodegenerative disorders. Frailty, sarcopenia, and polypharmacy present unique challenges, increasing risks of drug–nutrient interactions that may affect dietary intervention safety and feasibility [11]. These physiological and clinical features necessitate age-specific IF evaluation, as older adults present metabolic and functional vulnerabilities that could benefit from—or be adversely affected by—fasting-induced changes.

Although numerous trials and reviews have established IF benefits in younger populations [12,13,14,15], evidence for older adults remains limited [16]. Existing studies often feature small samples, short follow-up, heterogeneous designs, and lack aging-specific subgroup analyses [2,17]. Furthermore, comparative effects of different IF protocols in older adults have not been synthesized. To address these gaps, we conducted a systematic review and network meta-analysis (NMA) to evaluate IF safety and effectiveness in adults aged ≥60 years, focusing on anthropometric, cardiometabolic, mental health, and cognitive outcomes.

## 2. Materials and Methods

### 2.1. Study Design

The present systematic review and NMA were conducted in accordance with the Cochrane handbook for systematic reviews of interventions [18]; in addition, the Preferred Reporting Items for Systematic Reviews and Meta-analysis (PRISMA) [19] and the PRISMA extension statement for conducting NMA were used to report the results [20]. The systematic review encompassed all eligible study designs to provide a comprehensive contextual synthesis of the available evidence, while the NMA was restricted exclusively to RCTs, in accordance with the methodological requirements of network meta-analysis and the assumptions of transitivity and consistency. This systematic review has been registered in the International Prospective Register of Systematic Reviews (PROSPERO) [21] with the registration number CRD420251068135 (approved date 10 June 2025).

### 2.2. Data Sources and Search Strategy

A systematic search was performed in MEDLINE (PubMed), Scopus and ScienceDirect until June 2025, complemented by the manual screening of reference lists. The search combined MeSH terms and keywords for: population (adults ≥60 years: “older adults,” “elderly,” “aged”), interventions (IF modalities: “intermittent fasting,” “time-restricted eating,” “alternate-day fasting”), and outcomes (clinical variables, anthropometric measures, mental health and cognitive indicators). Searches were limited to English and Spanish publications without follow-up restrictions. Two researchers (SC and CS) independently conducted the search and screening, with discrepancies resolved by a third reviewer (II).

**Figure 1:**
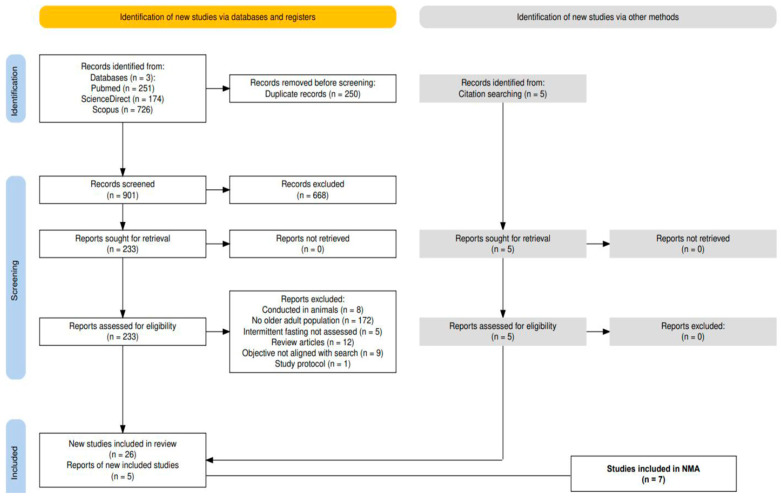
PRISMA flow diagram.

Studies were selected using PICOS criteria (Table 1). Exclusions comprised animal studies, those not reporting specified outcomes, and studies primarily in populations <60 years. Duplicates were removed manually in Excel, followed by title/abstract screening and full-text review with documented exclusion reasons. A PRISMA flow diagram was created to summarize the number of records identified, screened, assessed for eligibility and included (Figure 1). Multiple publications from the same cohort were treated as single studies, prioritizing the most comprehensive dataset.

### 2.3. Data Extraction

Two reviewers (SC and CS; 80% agreement) independently extracted data on study characteristics (author, year, country, design, sample size, and duration), participant features (age, sex, and health status), intervention details (IF type, control group, and fasting protocol), and outcomes (anthropometric, mental health, cognitive variables, dietary assessment, adjustments, and results). Discrepancies were resolved by consensus or a third reviewer (II).

For continuous outcomes, mean differences (MDs) and standard errors/deviations were extracted, prioritizing change from baseline over end-of-study values. Data were digitized from graphs when necessary, using WebPlotDigitizer. One eligible RCT used a crossover design; for this study, within-subject mean differences and standard errors from mixed-effects analyses were extracted, as recommended by the Cochrane Handbook for crossover trials.

Primary outcomes included body weight, depression, anxiety and cognitive function. Secondary outcomes encompassed anthropometric measures, glycemic and insulin markers, blood pressure, lipid profile, liver function, inflammatory markers and adverse events. Variables reported in <3 studies were excluded from NMA. Outcome units were standardized across studies.

### 2.4. Quality Assessment

Risk of bias was assessed using RoB 2.0 (RCTs) and ROBINS-I (non-randomized studies) by two independent reviewers (SC and CS), with disagreements resolved through discussion or adjudication. RoB 2.0 is the Cochrane tool for evaluating bias in randomized trials across domains such as randomization, deviations from intended interventions and outcome measurement, whereas ROBINS-I is the corresponding tool designed to assess bias in non-randomized studies by comparing them to a hypothetical target randomized trial. Detailed results of these assessments are presented in Supplementary Material S3.

### 2.5. Statistical Analysis

Analyses followed Cochrane [18] and NMA guidelines [20]. Seven studies were included. A frequentist NMA compared IF protocols (TRE, ADF, and 5:2) and controls for body weight and BMI; limited data precluded NMA for other outcomes. Sensitivity analyses restricted to RCTs and sequential exclusion identified outliers. Network geometry, league tables and P-scores evaluated comparative effectiveness. Consistency was assessed through global design-by-treatment approaches.

MD or SMD with 95% CI were calculated for continuous outcomes, using adjusted estimates when available, where MD represents absolute mean differences and SMD standardized mean differences across studies with different scales. Positive values indicated beneficial IF effects. Pairwise random-effects meta-analyses (DerSimonian–Laird) estimated direct comparisons between two interventions while accounting for between-study heterogeneity. Heterogeneity was quantified using Q (overall heterogeneity test), *I*^2^ (percentage of variability due to heterogeneity rather than chance) and τ^2^ (between-study variance), with prediction intervals used to assess the expected range of effects in future studies [22]. Treatment ranking used SUCRA values (0–1 scale, where higher values indicate higher probability of being among the best options) and mean ranks to summarize the comparative performance of each intervention across the network [23].

Sensitivity analyses excluded high-risk and non-randomized studies, and compared fixed versus random-effects models. Publication bias was explored with funnel plots (Supplementary Material S2: Figures S1 and S2); Egger’s test was not performed (<10 studies). Confidence in results was evaluated using the CINeMA framework [24] adapted from GRADE [25], assessing within-study bias, reporting bias, indirectness, imprecision, heterogeneity and incoherence.

Transitivity was assessed a priori by examining the distribution of potential clinical and methodological effect modifiers—including mean age, baseline body weight and BMI, proportion of participants with metabolic comorbidities, sex ratio, and intervention duration—across all pairwise treatment comparisons forming the network. Given that all NMA-eligible studies enrolled adults aged ≥60 years with comparable population characteristics and used usual diet or healthy living diet as comparators, the transitivity assumption was considered reasonably satisfied. Residual heterogeneity across studies was further addressed by retaining a random-effects model and is acknowledged in the certainty-of-evidence ratings. Analyses used R (version 4.5.1) with netmeta and metafor packages.

## 3. Results

### 3.1. Study Selection and Characteristics

Of the 31 studies meeting the inclusion criteria, 9 were RCTs [26,27,28,29,30,31,32,33,34], 12 were pre–post clinical trials without control groups [35,36,37,38,39,40,41,42,43,44,45,46], and 10 were observational cross-sectional or cohort studies [47,48,49,50,51,52,53,54,55,56]. Of these, only seven RCTs [26,27,28,29,30,33,34] satisfied the eligibility criteria for inclusion in the NMA. Pre–post and observational studies contributed exclusively to the narrative synthesis. Additionally, NMA was feasible only for body weight (Section 3.4.1) and BMI (Section 3.4.2), as insufficient data precluded network synthesis for other outcomes. Section 3.3 presents a structured narrative synthesis of findings from individual studies, organized by outcome domain. A PRISMA flow diagram was created with the Shiny app [57], which is an interactive web application created in R, to represent the screening process of articles found in the bibliographic search (Figure 1).

Sample sizes ranged from 9 to over 2.2 million participants. Most interventions tested TRE (TRE, typically 16:8 or 14:10), followed by 5:2 IF regimens and Ramadan fasting. Populations were heterogeneous, including healthy older adults, individuals with obesity, metabolic syndrome, cardiovascular risk, rheumatoid arthritis, chronic obstructive pulmonary disease, or mild cognitive impairment. The mean age across studies was above 60 years. Intervention durations varied between 4 and 12 weeks for clinical trials and up to 3 years for cohort studies. Studies were carried out in different countries, such as Poland [27,28], Malaysia [33,34,48,49], the USA [29,30,31,36,38,40,44,45,46], Spain [26,53,54], Iran [32], Tunisia [35,37,39,42,43], Australia [41], China [52,55,56], South Korea [50] and Italy [47,51]. Main characteristics and results are shown in Table 2, Table 3, Table 4, Table 5 and Table 6.

### 3.2. Risk of Bias and Study Quality

Risk of bias among RCTs ranged from low to moderate (Supplementary Material S3: Table S1). Four studies [26,30,31,32] were judged as low risk, whereas five others [27,28,29,33,34] were rated as having “some concerns,” primarily related to randomization procedures and selective reporting. Non-randomized studies generally showed fair to moderate quality on the NIH assessment tool, with higher quality observed in prospective cohorts [48,49,55,56] (Supplementary Material S4, Tables S2–S4). Sensitivity analyses restricting the NMA to RCTs only reduced heterogeneity and confirmed the overall direction of effects.

### 3.3. Results of the Systematic Review

#### 3.3.1. Anthropometric and Body Composition Outcomes

Ten studies [27,28,30,33,34,38,40,41,45,48] evaluated IF effects on body weight, reporting reductions from −1.36 to −3.65 kg across different modalities (primarily TRE and IF 5:2).

TRE 16:8 studies showed consistent weight loss with muscle mass preservation. Short-term RCTs (6 weeks) in healthy older women [27] and overweight men [28] demonstrated weight loss of −1.36 kg and −1.50 kg, respectively, with BMI reductions of −1.29 and −0.50 kg/m^2^. The latter also showed a waist circumference reduction of −2.8 cm and decreased visceral fat without lean mass loss. A 4-week pre–post study in sedentary overweight adults [45] reported −2.15 ± 1.43 kg weight loss and −0.9 ± 0.6 kg/m^2^ BMI reduction with high adherence and minimal adverse events.

Longer-duration interventions showed more pronounced effects. A 12-week 14:10 TRE study in adults with metabolic syndrome [38] achieved −3.30 ± 3.20 kg weight loss, −1.09 ± 0.97 kg/m^2^ BMI reduction, and −4.46 cm waist circumference decrease with reductions in total and visceral fat. Another 12-week RCT comparing 8–10 h TRE to Mediterranean diet [30] showed −2.98 kg weight loss and −1.11 kg/m^2^ BMI reduction in the TRE group, significantly outperforming controls (−1.32 kg; *p* < 0.05), with decreased trunk fat and preserved lean mass.

The most notable finding comes from a 3-year prospective cohort [48]: older adults with mild cognitive impairment practicing regular ISF lost −3.65 kg with −1.53 kg/m^2^ BMI reduction and −3.57 cm waist circumference decrease, while non-fasters showed biomarker deterioration and weight-gain tendency.

IF 5:2 combined with caloric restriction also proved effective. Two 12-week studies in older men [33,34] using adapted ISF (two partial fasting days with 300–500 kcal plus five days of moderate restriction) reported weight losses of −2.80 kg and −2.5 kg with BMI reductions of −1.00 and −0.9 kg/m^2^, respectively, both with body fat percentage reductions.

Short-term interventions (4–8 weeks) demonstrated effectiveness even without controls: a 4-week TRE 16:8 study [40] showed −2.6 kg weight loss, while an 8-week study in older men with obesity [41] found −2.3 kg weight loss, −0.7 kg/m^2^ BMI reduction, and −4 cm waist circumference decrease with reduced visceral fat despite unchanged total caloric intake.

#### 3.3.2. Metabolic and Cardiovascular Outcomes

Thirteen studies [26,29,30,31,32,33,38,41,48,49,53,55,56] evaluated IF effects on metabolic and cardiovascular variables, reporting heterogeneous but generally favorable results.

Glycemic control improved across multiple trials. A 12-week RCT [30] showed 8–10 h TRE reduced HbA1c by −0.12% versus the Mediterranean diet. An 8-week 14:10 TRE study [41] decreased fasting glucose (−0.3 mmol/L) and HbA1c (−0.2%) with improved postprandial glucose-dependent insulinotropic peptide responses. The 3-year ISF cohort [48] demonstrated −0.37 mmol/L fasting glucose reduction and marked insulin decrease (−16.25 pmol/L) in fasters versus metabolic deterioration in non-fasters. An 8-week 5:2 IF RCT [29] showed improvements in neuronal insulin resistance, with slightly greater effects in IF versus healthy-living diet.

Lipid profiles showed mixed results. The 12-week TRE trial in metabolic syndrome [38] significantly decreased total cholesterol (−13.16 mg/dL), LDL-c (−11.94 mg/dL), and non-HDL cholesterol (−11.63 mg/dL). However, a 6-week TRE 16:8 RCT in healthy adults [31] reported modest increases in total cholesterol (+11 mg/dL) and LDL-c (+11 mg/dL) without adverse clinical effects. Observationally, skipping dinner was associated with lower LDL-c and triglycerides versus skipping breakfast [50], while prolonged nightly fasting (≥12 h) was linked to reduced HDL-c (−2.79 mg/dL; *p* = 0.01) [53].

Blood pressure consistently improved. A 12-week metabolic syndrome TRE trial [38] reduced systolic (−5.12 mmHg) and diastolic pressure (−6.47 mmHg). TRE combined with the Mediterranean diet [26] achieved greater systolic reduction (−9.8 mmHg) versus diet alone (−13.3 mmHg in controls, with between-group differences favoring TRE for anthropometrics). A 12-week ISF + caloric restriction intervention [33] lowered systolic (−6.5 mmHg) and diastolic blood pressure (−2.2 mmHg). A 3-year cohort [48] showed −7.04 mmHg systolic blood pressure reduction in regular fasters versus no benefit in non-fasters.

Oxidative stress and inflammation markers improved notably. An 8-week TRE 16:8 RCT in postmenopausal women with rheumatoid arthritis [32] significantly reduced AST (−4.82 U/L), ALT (−2.34 U/L), MDA (−0.01 µM), and neutrophil-to-lymphocyte ratio (−0.11), while increasing catalase activity (+0.02 µM). A 3-year ISF cohort [48,49] reported MDA and C-reactive protein reductions with increased SOD activity in fasters—effects absent in non-fasters.

Arterial health and cardiovascular risk yielded complex results. A Chinese cross-sectional study [55] found that TRE with an eating window ≤11 h was associated with higher odds for arterial stiffness (OR = 1.70; 95% CI: 1.28–2.26), particularly in those at risk for malnutrition. Conversely, a U.S. NHANES prospective cohort [56] revealed a U-shaped relationship: night-time fasting >12.38 h was associated with 58% higher cardiovascular death risk (HR = 1.58; 95% CI: 1.10–2.28) versus intermediate durations (~11.5 h), which seemed to be the safest.

#### 3.3.3. Mental Health Outcomes

Eight studies [34,35,36,43,46,51,52,54] examined IF effects on mental health in older adults, assessing mood, anxiety, depression, insomnia, and mental distress using validated instruments (POMS, GAD-7, GDS, and ISI).

Structured IF protocols improved mood and emotional well-being. A 12-week ISF + caloric restriction RCT [34] showed significant POMS reductions in tension (−1.0), anger (−1.9), and confusion (−2.0), with increased vigor (+1.6) and decreased total mood disturbance (−10.4).

Ramadan fasting produced mixed results. One pre–post study [43] found significant anxiety (GAD-7: −3) and depression (GDS: −1) reductions in both active and sedentary participants, but a similar 2022 study [35] found no significant changes despite physical performance improvements, suggesting physical activity’s moderating role.

Sleep quality varied by protocol. An 8-week 14:10 nightly TRE pilot study [36] in adults with memory decline reduced insomnia severity (ISI: −1.72), suggesting circadian-aligned eating windows may improve sleep. Conversely, Ramadan fasting worsened sleep quality in both active and sedentary groups [35].

Observational data revealed contrasting patterns. Italian older adults with ≤8 h eating windows had 86% lower mental distress odds (OR = 0.14; 95% CI: 0.03–0.65), independent of Mediterranean diet adherence and sociodemographic data [51]. However, a Spanish cohort showed that those fasting ≥12 h overnight had significantly higher clinical depression prevalence versus ≤9 h fasters, even after adjusting for confounders [54].

Two studies found no significant effects: a 4-week TRE intervention [46] reported high satisfaction, but no measurable mood changes, and a Chinese cross-sectional study [52] found no association between 14:10 TRE and depression or sleep duration.

#### 3.3.4. Cognitive Outcomes

Fourteen studies [28,29,31,35,36,37,39,43,45,46,47,48,49,52] examined IF effects on cognition. Across included studies, cognitive function was operationalized using a range of validated standardized neuropsychological instruments: MMSE, MoCA, RAVLT, MAPS, reaction time measures and the NIH Toolbox Cognition Battery. These instruments assess distinct cognitive domains, including global status (MMSE and MoCA), episodic memory (RAVLT), processing speed (Digit Symbol test) and executive function. None of the included studies used clinically diagnosed dementia incidence as an outcome; all cognitive findings reflect measurable changes in test performance. Benefits emerged primarily in structured, long-term, or circadian-aligned protocols, while short-term or disruptive fasting showed mixed results.

Structured, circadian-aligned IF consistently improved cognition. An 8-week 5:2 IF RCT [29] significantly enhanced executive function, fluency, set-shifting, cued recall, and global performance, reducing “brain age” by 2.63 years versus healthy-living diet controls. An 8-week nightly 14:10 TRE pilot [36] (eating window ending by 8 PM) improved global cognitive function (+11.88 MAPS points) in adults with memory decline. Cross-sectional Italian data [47] showed TRE practitioners (≤10 h window) had 72% lower cognitive impairment odds (OR = 0.28; 95% CI: 0.07–0.90), particularly with breakfast inclusion.

Long-term regular fasting yielded the most robust benefits. The 3-year prospective cohort [48] in adults with mild cognitive impairment showed significant improvements across all domains in regular ISF practitioners: MMSE (+6.43), MoCA (+5.16), RAVLT (+6.05), and processing speed (+1.28 Digit Symbol), while non-fasters declined markedly. Follow-up analysis [49] confirmed regular fasters scored significantly higher than non-fasters on MMSE (24.05 vs. 16.33), MoCA (19.43 vs. 14.37), and memory tests.

Ramadan IF produced activity-dependent outcomes. Physically active participants showed improved executive function, attention, inhibition, memory [35], and reduced vigilance reaction time [43]. However, sedentary participants experienced declined associative learning [35], and both groups showed worsened simple reaction time during weeks 2 and 4 [37,39].

Short-term or unstructured TRE generally showed no effects. A 4-week TRE 16:8 intervention [45] and two 6-week RCTs [28,31] found no MoCA or MMSE changes in sedentary overweight adults, healthy women, and overweight men, respectively. A 4-week feasibility study [46] also reported no cognitive changes.

Notably, very short eating windows were associated with worse performance. In a Chinese cohort [52], older adults consuming all calories within ≤10 h had significantly lower MMSE scores (22.45 vs. 24.97; *p* < 0.001), particularly in orientation and attention/calculation domains.

### 3.4. Results of the Network Meta-Analysis

#### 3.4.1. Body Weight (kg)

**Figure 2:**
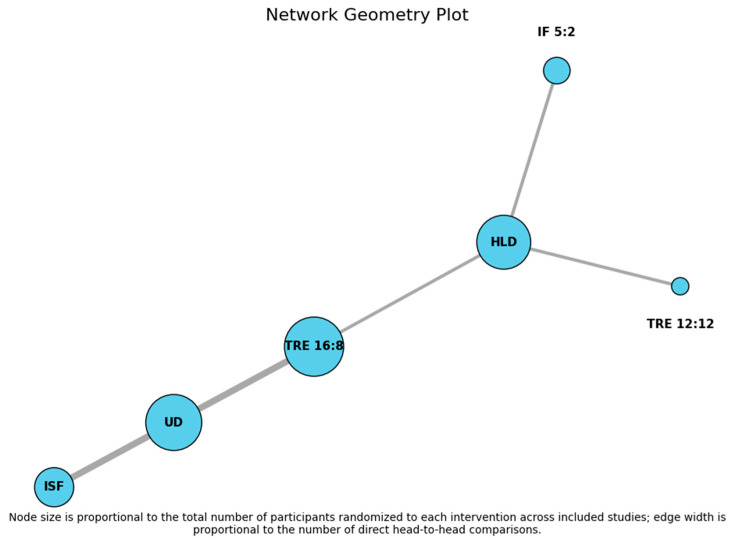
Network geometry plot.

A network meta-analysis (NMA) including seven randomized studies compared six dietary interventions (HLD, IF 5:2, ISF, TRE 12:12, TRE 16:8, and usual diet [UD] as reference) for body weight reduction. In Figure 2, we present the network geometry plot, which is a graphical map of the network meta-analysis showing each intervention as a node and the available direct comparisons between interventions as connecting lines, with line thickness reflecting the amount of evidence. All seven studies included in the NMA were RCTs, ensuring that the pooled estimates and treatment rankings are derived exclusively from experimental, randomized evidence.

No evidence of heterogeneity was observed across the network (Total Q = 0.26, *df* = 2, *p* = 0.88). Heterogeneity within designs was also negligible (Q = 0.26, *df* = 2, *p* = 0.88), indicating that results were highly consistent among studies comparing the same interventions. Design-specific analyses confirmed the absence of heterogeneity for the direct comparisons: ISF vs. UD (Q = 0.26, *p* = 0.61) and TRE 16:8 vs. UD (Q = 0.00, *p* = 0.99).

Assessment of global inconsistency through design-by-treatment interaction could not be performed due to the absence of closed loops in the network, which is expected in networks with a star-shaped structure. Finally, tau^2^ was estimated as 0, further supporting the extreme homogeneity among studies.

The pooled effect estimate indicated a significant overall reduction in body weight (MD = −2.04 kg; SE = 0.22; 95% CI −2.47 to −1.61; *p* < 0.001). Given the absence of between-study heterogeneity, the prediction interval was narrow and overlapped the confidence interval (−2.61 to −1.47). A random-effects model was retained to account for potential clinical and methodological variability, in accordance with PRISMA-NMA recommendations.

Using UD as the reference, ISF and TRE 16:8 demonstrated statistically significant weight reductions with precise estimates (ISF: MD = −2.36 kg, 95% CI −2.93 to −1.79; TRE 16:8: MD = −1.92 kg, 95% CI −2.57 to −1.27; both *p* < 0.0001). In contrast, IF 5:2, HLD, and TRE 12:12 showed no clear evidence of benefit over UD, as confidence intervals crossed the null effect (Table 7). In this network of seven studies, rankings were inherently unstable and should be interpreted with caution; small changes in the included evidence could substantially alter ranking order.

**Figure 3:**
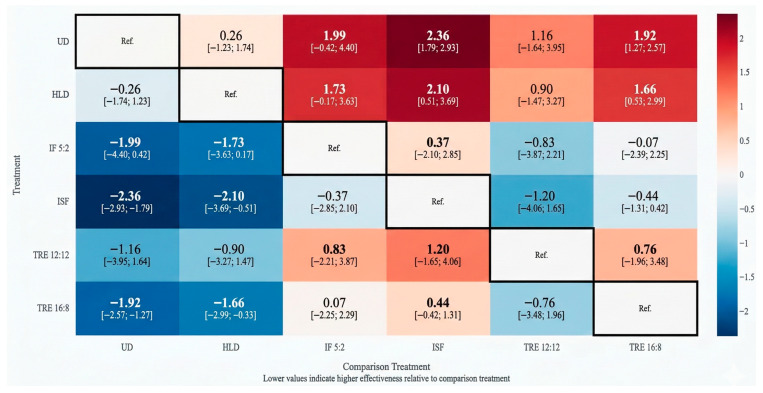
League table of pairwise comparisons for weight outcomes.

Figure 3 presents all pairwise mean differences (95% CIs) for weight for every treatment comparison within the network, including both direct and indirect estimates, with certainty ratings per comparison. In the color scale, blue cells indicate comparatively more beneficial effects (greater weight reduction relative to the reference treatment), whereas red cells indicate comparatively less beneficial or unfavorable effects.

Ranking probabilities based on P-scores identified ISF as the most effective intervention (0.850), followed by IF 5:2 (0.704) and TRE 16:8 (0.667). TRE 12:12 (0.471) and HLD (0.182) ranked lower, while UD showed the lowest probability of effectiveness (0.126). These rankings should be interpreted alongside effect sizes and certainty of evidence (Table 8).

Clinical imprecision was assessed using a prespecified threshold of ±3.5 kg (5% of a reference body weight of 70 kg) [58,59], consistent with clinically meaningful weight loss. Two studies presented serious imprecision, with confidence intervals crossing this threshold [26,29], whereas the remaining studies showed low imprecision [27,28,30,33,34], supporting the clinical relevance of the observed effects.

All included studies contributed direct evidence to their respective comparisons, and no study relied solely on indirect evidence. Although some treatment contrasts were informed only indirectly at the network level, concerns regarding indirectness were considered minor and did not affect the certainty of evidence for individual comparisons. Visual inspection of funnel plots revealed no clear asymmetry, suggesting a low risk of publication bias (Supplementary Material S2).

#### 3.4.2. BMI (kg/m^2^)

The network meta-analysis compared six dietary interventions (HLD, IF 5:2, ISF, TRE 12:12, TRE 16:8, and usual diet [UD] as reference). All seven studies included in the NMA were RCTs, ensuring that the pooled estimates and treatment rankings are derived exclusively from experimental, randomized evidence.

The network showed evidence of heterogeneity (Total Q = 6.95, *df* = 2, *p* = 0.031), indicating some variability among the included studies. Heterogeneity within designs was similar (Q = 6.95, *df* = 2, *p* = 0.031). Design-specific analyses indicated that heterogeneity was present for TRE 16:8 vs. UD (Q = 6.48, *p* = 0.011), while ISF vs. UD showed no evidence of heterogeneity (Q = 0.47, *p* = 0.49).

Global inconsistency could not be assessed due to the absence of closed loops in the network. Estimated tau within designs was 0.3981, and tau^2^ within designs was 0.1585, reflecting moderate heterogeneity among studies.

The pooled random-effects estimate showed a statistically significant reduction in BMI (MD = −0.77 kg/m^2^; SE = 0.09; z = −8.48; *p* < 0.001), with a narrow 95% confidence interval (−0.95 to −0.59) and a nearly identical prediction interval (−0.97 to −0.57), reflecting substantial homogeneity of effects.

P-score ranking placed TRE 16:8 as the most effective intervention (0.751), followed by IF 5:2 (0.729) and ISF (0.618). TRE 12:12 (0.499) and HLD (0.259) showed lower probabilities of benefit, while UD ranked lowest (0.143). Notably, IF 5:2 achieved a relatively high ranking despite lacking statistical significance versus UD (Table 8).

**Figure 4:**
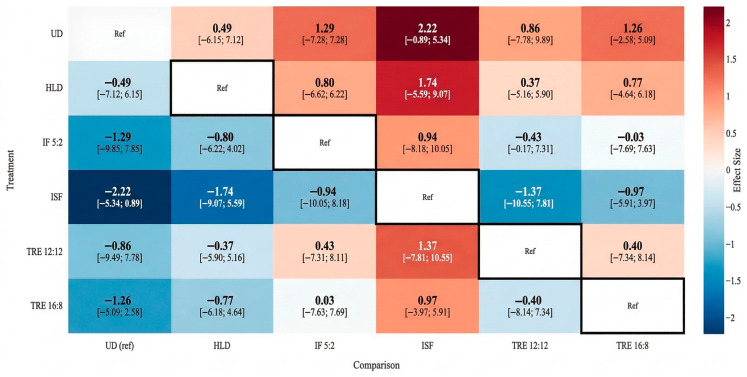
League table of pairwise comparisons for BMI outcomes.

Using UD as the comparator, TRE 16:8 and ISF demonstrated statistically significant BMI reductions (TRE 16:8: MD = −1.01 kg/m^2^, 95% CI −1.69 to −0.33, *p* = 0.0037; ISF: MD = −0.81 kg/m^2^, 95% CI −1.43 to −0.19, *p* = 0.0106). IF 5:2, HLD, and TRE 12:12 did not differ significantly from UD, as their confidence intervals crossed the null effect (Table 9). Figure 4 presents all pairwise mean differences (95% CIs) for BMI for every treatment comparison within the network, including both direct and indirect estimates, with certainty ratings per comparison.

Clinical imprecision was evaluated using a prespecified threshold of ±1.25 kg/m^2^, corresponding to a 5% weight loss for a reference individual (70 kg, 1.70 m) [58,59]. Five studies [26,27,28,29,30] exhibited serious imprecision, with confidence intervals crossing this threshold, whereas two studies [33,34] showed moderate imprecision, with lower bounds close to the cutoff.

All included studies contributed direct evidence through pairwise comparisons, and no trial relied exclusively on indirect evidence. Although some treatment contrasts were informed only indirectly at the network level, concerns regarding indirectness were considered minor and did not affect individual study certainty. Funnel plot inspection suggested possible publication bias driven by one small study [27], which appeared as an outlier with a high standard error (Supplementary Material S2).

## 4. Discussion

This systematic review and NMA evaluated the safety and effectiveness of IF in adults aged ≥60 years. Our findings suggest that IF is generally feasible and appears to have an acceptable short-term safety profile in older adults, with modest but clinically meaningful benefits. ISF and TRE 16:8 showed the strongest comparative effects for body weight reduction (MD: −2.36 kg and −1.92 kg, respectively) and BMI reduction in the RCT-based NMA. Preservation of lean muscle mass was consistently reported across the included studies evaluating these protocols in the systematic review. Improvements were also observed in HbA1c, systolic blood pressure, and LDL cholesterol. However, evidence regarding mental health and cognitive outcomes remains inconclusive and highly heterogeneous.

The quantitative scope of the NMA is limited to body weight and BMI, for which sufficient RCT data were available. All other outcomes discussed in this review reflect narrative synthesis and must not be interpreted as comparative network estimates.

### 4.1. Anthropometric and Body Composition Outcomes

Weight loss documented across our studies (−1.36 to −3.65 kg) aligns closely with meta-analyses in mixed populations [60,61,62,63], suggesting IF’s mechanisms extend beyond age barriers. Our results parallel Semnani-Azad et al.’s findings (−3.40 kg for ADF, −1.72 kg for TRE) [62] and Chen et al.’s data in women (−1.927 kg) [61], indicating comparable responses across age groups.

It seems that intervention duration is critical: our 3-year prospective study [48] documenting −3.65 kg represents one of the longest follow-ups in geriatric literature, with interventions ≥24 weeks showing more sustained effects [62]. TRE 16:8 consistently demonstrated high adherence rates (>80%) with minimal adverse events, whereas more restrictive protocols showed greater variability [64,65,66,67].

Systematic preservation of muscle mass in TRE studies holds particular relevance given the risk for sarcopenia in older adults [68,69]. TRE 16:8 studies consistently reported the absence of lean mass loss [27,28,30], contrasting favorably with traditional continuous caloric restriction [70]. An umbrella review documented significant increases in fat-free mass (MD = 0.98 kg; 95% CI: 0.18–1.78) [63], likely reflecting preferential adipose tissue reduction.

Reductions in waist circumference (−2.8 to −4.46 cm) exceed clinically significant thresholds for cardiometabolic risk reduction [71], with effects particularly pronounced in combined 5:2 ISF protocols. The preferential loss of visceral fat—more metabolically active and proinflammatory—carries important metabolic implications in older adults [72,73].

Effects were more pronounced in populations with metabolic syndrome than in healthy individuals [74,75], suggesting specific therapeutic potential in those with geriatric comorbidities. These patterns imply complex metabolic adaptations, including improvements in insulin sensitivity and optimization of circadian rhythms related to lipid metabolism [76,77,78,79,80,81].

### 4.2. Metabolic and Cardiovascular Outcomes

Glycemic improvements with TRE, particularly HbA1c reductions, are comparable to meta-analyses in younger populations and fall within clinically relevant ranges for type 2 diabetes prevention [82,83,84,85]. TRE emerged as the most effective modality for fasting glucose reduction [62,83,86,87], likely through improvements in insulin sensitivity and pancreatic function optimization.

Lipid profile effects showed greater variability, with some trials documenting improvements in cholesterol and LDL-c while others reported modest increases in short TRE studies [75]. Blood pressure reductions were more consistent (−5.12 to −13.3 mmHg systolic) across different modalities [88,89].

Reductions in inflammatory markers (MDA, CRP, neutrophil–lymphocyte ratio) with increases in catalase and superoxide dismutase suggest robust anti-inflammatory effects. A network meta-analysis documented significant reductions in TNF-α (SMD: −0.31), CRP (SMD: −0.19), and leptin (SMD: −0.57) [90]. TRE appeared to cause the greatest TNF-α reductions (−0.39), particularly relevant given chronic low-grade inflammation’s role in functional decline and age-related diseases [91].

Concerning findings emerged from observational studies: nocturnal fasting >12.38 h was associated with a 58% higher cardiovascular mortality, while eating windows ≤11 h were linked to greater arterial stiffness. Taken together, these patterns are consistent with the possibility of a non-linear (U-shaped) relationship between fasting duration and cardiovascular outcomes, in which intermediate fasting intervals (around 11.5 h) may be comparatively safer, but this remains speculative. Mechanisms may involve adverse effects on heart rate variability, autonomic function, or medication interactions. However, these findings (e.g., cardiovascular mortality risk with prolonged nocturnal fasting, HR = 1.58; 95% CI: 1.10–2.28) derive exclusively from observational studies and are subject to inherent limitations, including residual and unmeasured confounding. Accordingly, these associations should be regarded as hypothesis-generating and interpreted with great caution, rather than as evidence of a causal effect.

### 4.3. Mental Health and Cognitive Outcomes

Mental health results reveal marked dependence on protocol structure. Circadian rhythm-aligned protocols (nocturnal TRE 14:10 or regular ISF) showed consistent benefits in mood, anxiety, and depression. Conversely, observational studies documented 86% lower mental distress in eating windows ≤8 h [51] but higher depression prevalence with nocturnal fasts ≥12 h [92], underscoring the importance of circadian alignment.

Cognitive effects present a complex pattern. Long-duration structured protocols showed robust benefits, particularly our 3-year study documenting improvements across all dimensions (MMSE +6.43, MoCA +5.16, RAVLT +6.05). However, a non-linear relationship emerged: while 8–10 h windows associated with better function, windows ≤10 h paradoxically linked to lower MMSE scores [52]. Ramadan studies suggest physical activity significantly modulates cognitive effects [35].

### 4.4. Clinical Implications for Older Adults

Mechanisms underlying observed effects involve metabolic flexibility through switching between substrate utilization [93], particularly relevant given age-related metabolic decline. Anti-inflammatory effects may additionally mitigate inflammaging [91].

Clinical implementation requires careful individualization. Initial protocols should be less restrictive (14:10 or 16:8), allowing gradual adaptation. Extremely restrictive windows (<8 h) should be avoided, given cardiovascular risk evidence. Circadian alignment should be prioritized over extreme restriction.

Integration with geriatric considerations is essential: adequate protein intake to prevent sarcopenia, monitoring of medication interactions, consideration of social eating aspects, and screening for malnutrition risk. Specific risks include dehydration, orthostatic hypotension, electrolyte disturbances, hypoglycemia in diabetics, and side effects that potentially increase fall risk.

### 4.5. Limitations and Future Directions

Most RCTs lasted <24 weeks, limiting conclusions about long-term sustainability. Publication bias may inflate effectiveness estimates. We could not assess IF interactions with medications, particularly relevant in this population.

Intervention heterogeneity within network nodes represents a relevant source of uncertainty. As the evidence base expands, future NMAs should disaggregate IF protocols with greater granularity, distinguishing protocols by fasting duration, eating window timing, caloric restriction depth, and circadian alignment.

In the context of Islamic Sunnah fasting, spiritual and faith-based practices (e.g., communal worship, structured routines, stress reduction, and sense of purpose) may also contribute to health benefits, independently of or alongside metabolic effects of IF. However, the included ISF studies did not assess religiosity or spiritual well-being, so these influences could not be disentangled. Future research on religious fasting should incorporate such measures to clarify their role.

Future research should prioritize longer-duration trials with longitudinal muscle mass evaluations through advanced imaging, sarcopenia biomarkers, functional outcomes, and systematic adverse event monitoring. Studies examining IF interactions with lifestyle modifiers are needed.

## 5. Conclusions

This systematic review and network meta-analysis evaluated IF protocols in adults aged ≥60 years. Moderate-certainty evidence from seven RCTs suggests that TRE 16:8 and ISF produce statistically significant and clinically meaningful reductions in body weight (ISF: −2.36 kg, 95% CI −2.93 to −1.79; TRE 16:8: −1.92 kg, 95% CI −2.57 to −1.27) and BMI compared to usual diet, with minimal heterogeneity in the networks (weight: Q = 0.26, *p* = 0.88; BMI: Q = 6.95, *p* = 0.03).

The systematic review suggests these protocols are associated with improvements in metabolic markers (HbA1c and blood pressure) and preservation of lean muscle mass across included studies, though comparative quantitative evidence is limited to anthropometric outcomes. Mental health and cognitive findings derive exclusively from narrative synthesis of heterogeneous study designs and remain exploratory.

Future trials should be long-term, statistically well-powered, and age-specific, directly comparing protocols while accounting for modulators such as frailty, comorbidities, circadian phenotype, and polypharmacy. Personalization of IF regimens and evaluation of long-term safety are essential to develop evidence-based dietary strategies for healthy aging.

## Figures and Tables

**Table 1:** PICO elements used to define the research question.

Element	Description
Population (P)	Adults aged ≥60 years with or without chronic conditions (overweight/obesity, type 2 diabetes, and cardiometabolic risk).
Intervention (I)	Intermittent fasting protocols: time-restricted eating (TRE 16:8, TRE 12:12), 5:2 regimen, and Islamic Sunnah fasting (ISF).
Comparison (C)	Usual diet, healthy living diet, or control diets without time restriction.
Outcomes (O)	Anthropometric (weight, BMI, and lean mass), cardiometabolic (HbA1c, systolic blood pressure, and LDL cholesterol), mental health (depression, anxiety, and insomnia), cognitive function (changes in performance on validated neuropsychological instruments including MMSE, MoCA, RAVLT, MAPS and NIH Toolbox Cognition Battery, feasibility, and safety.
Study design (S)	For the NMA: Randomized controlled trials (RCTs) only. For the narrative systematic review: RCTs, pre–post clinical trials (single-arm), cohort studies, and cross-sectional studies.

**Table 2:** Main characteristics of the included interventional studies.

Author, Year	Country	Study Design	Nº (IG)	Age of Participants	Sexual Distribution	Type of Intervention	Control Group Intervention	Duration of Intervention	Quality Assessment
Couto et al., 2025 [26]	Spain	RCT	17 with NCD (8)	61–80 y	10 men; 7 women	TRE 12:12 + Mediterranean diet	Mediterranean diet	12 wks	64.3%
Domaszewski et al., 2020 [27]	Poland	RCT	42 healthy women (22)	≥60 y	Not specified	TRE 16:8	Usual diet	6 wks	64.3%
Kapogiannis et al., 2024 [29]	USA	RCT	40 healthy participants (20)	55–70 y	8 men; 24 women	IF 5:2 2 consecutive days: maximum 480 kcal/d; 5 days: healthy living diet	Healthy living diet 7 days/wk	8 wks	78.6%
Domaszewski et al., 2022 [28]	Poland	RCT	46 overweight men (23)	65–74 y	Not specified	TRE 16:8	Usual diet	6 wks	71.4%
Hussin et al., 2013 [34]	Malaysia	RCT	31 males (16)	50–70 y	Not specified	IF 5:2 2 days: Islamic Sunnah fasting (14–16 h approx.); 5 days: 300–500 kcal restriction	Usual diet	12 wks	64.3%
Teng et al., 2013 [33]	Malaysia	RCT	56 (28) healthy men	50–70 y	Not specified	IF 5:2 2 days: Islamic Sunnah fasting (14–16 h approx.); 5 days: 300–500 kcal restriction	Usual diet	12 wks	57.1%
Martens et al., 2020 [31]	USA	RCT	22 (12)	55–79 y	Not specified	TRE 16:8	Usual diet	6 wks	85.7
Tavakoli et al., 2025 [32]	Iran	RCT	44 PM, OW/OB women (22)	50–70 y	Not specified	TRE 16:8 with a 55% carbs, 30% fat, 15% protein and 300 kcal/day deficit diet.	Usual diet with recommendations for healthy eating	8 wks	57.1%
Manoogian et al., 2024 [30]	USA	RCT	108 with metabolic syndrome (54)	18–75 y (overall mean baseline age: 59 y)	60 men; 62 women	TRE 16:8	Mediterranean diet recommendations and healthy lifestyle advice	12 wks	58.7%
Boujelbane et al., 2022 [35]	Tunisia	Pre–post CT without CG	58	60–79 y	27 men; 31 women	Ramadan IF (physically active group)	Ramadan IF (sedentary group)	4 wks	72.7%
Saini et al., 2022 [40]	USA	Pre–post CT without CG	9 with OW	≥65 y	3 men; 6 women	TRE 16:8 with self-selected fasting/eating times	No CG	4 wks	63.6%
Zhao et al., 2022 [41]	Australia	Pre–post CT without CG	15 men with OB	40–70 y (overall mean baseline age: 63 y)	15 men	TRE 14:10	No CG	8 wks	63.6%
Mrad et al., 2019 [42]	Tunisia	3-measurement CT without CG	15 males with COPD	71 ± 6 years	15 men	Ramadan IF	No CG	4 wks	63.6%
Boujelbane et al., 2025 [43]	Tunisia	Pre–post CT without CG	58	≥60 y	27 men; 31 women	Ramadan IF (physically active group)	Ramadan IF (sedentary group)	4 wks	63.6%
Ezzati et al., 2025 [44]	USA	Pre–post CT without CG	10 OW people	≥65 y	Not specified	TRE 16:8 with self-selected fasting/eating times	No CG	4 wks	63.6%
Anton et al., 2019 [45]	USA	Pre–post CT without CG	10 OW and sedentary people	≥65 y	Not specified	TRE 16:8 with self-selected fasting/eating times	No CG	4 wks	63.6%
Wilkinson et al., 2020 [38]	USA	Pre–post CT without CG	19 people with metabolic syndrome	≥18 y (overall mean baseline age: 59 y)	Not specified	TRE 14:10 with self-selected fasting/eating times	No CG	12 wks	63.6%
Laatar et al., Nov 2016 [39]	Tunisia	3-measurement CT without CG	24 sedentary males	65–80 y	Not specified	Ramadan IF (fallers)	Ramadan IF (non-fallers)	4 wks	63.6%
James et al., 2024 [36]	USA	Pre–post CT without CG	18 people with memory decline	≥65 y	1 man; 19 women (initially)	TRE 14:10 with night fasting (after 8PM), 6 days/wk	No CG	8 wks	63.6%
Laatar et al., May 2016 [37]	Tunisia	4-measurement CT without CG	15 sedentary men	65–80 y	Not specified	Ramadan IF	No CG	4 wks	63.6%
Lee et al., 2020 [46]	USA	Pre–post CT without CG	10 OW, sedentary people with functional limitations	≥65 y	Not specified	TRE 16:8 with self-selected fasting/eating times	No CG	4 wks	63.6%

RCT: Randomized controlled trial; CT: clinical trial; CG: control group; IG: intervention group; TRE: time-restricted eating; IF: intermittent fasting; 16:8: 16 h fasting period, 8 h eating window; PM: postmenopausal; OW: overweight; OB: obese; wks: weeks; y: years.

**Table 3:** Main characteristics of the observational included studies.

Author, Year	Country	Study Design	Study Name	Nº	Age of Participants	Sex Distribution	Exposure	Exposure Classification	Quality Assessment
Currenti et al., Jun 2021 [51]	Italy	Cross-sectional	MEAL study	1572 Subgroup ≥70 y: n = 174	≥70 y	660 men; 912 women	TRE	- 16:8 - No TRE	66.7%
Li et al., 2023 [52]	China	Cross-sectional	-	1353	≥60 y	563 men; 790 women	TRE	- Eating window: - <10 h - >10 h	66.7%
Chen et al., 2024 [55]	China	Cross-sectional	-	3487	≥60 y	Not specified	TRE	- Eating window: - <11 h - >11 h	66.7%
Estrada-deLeón et al., 2021 [54]	Spain	Cross-sectional	Seniors-ENRICA-II	1226	≥64 y	628 men; 598 women	Prolonged nightly fasting	- ≤9 h - 10–11 h - ≥12 h	66.7%
Estrada-deLeón et al., 2022 [53]	Spain	Cross-sectional	Seniors-ENRICA-2	1047	≥65 y	537 men; 510 women	Prolonged nightly fasting	- ≥12 h - <10 h - 10–<12 h	75%
Currenti et al., Jan 2021 [47]	Italy	Cross-sectional	MEAL study	883	≥50 y (overall mean baseline age: 65.1 y)	Not specified	TRE	- Eating window: - <10 h - >10 h	66.7%
Kang et al., 2024 [50]	South Korea	Cross-sectional	KNHANES 2013–2020	28,530 Subgroup ≥65 y: n = 8349	≥65 y	19074 men; 15444 women	Meal skipping	- No skipping - Skipping breakfast (<2/wk) - Skipping dinner (<2/wk)	66.7%
Ooi et al., 2020 [48]	Malaysia	Prospective cohort study, 3 y duration	LRGS-TUA	99 with mild cognitive impairment	≥60 y	53 men; 46 women	Islamic Sunnah IF	- Regular IF - Irregular IF - Non-IF	92.9%
Zhang et al., 2024 [56]	USA	Prospective cohort study, median: 6.66 y	NHANES 2005–2018	10,561	≥60 y	5821 men; 4040 women	Night-time fasting	- ≤7.5 h - >7.5–10.58 h - >10.58–12.38 h - >12.38 h	92.9%
Ooi et al., 2022 [49]	Malaysia	Prospective cohort study, 3 y duration	LRGS-TUA	99 with mild cognitive impairment	≥60 y	53 men; 46 women	Islamic Sunnah IF	- Regular IF - Irregular IF - Non-IF	92.9%

TRE: Time-restricted eating; IF: intermittent fasting; 16:8: 16 h fasting period, 8 h eating window; wk: weeks; y: years.

**Table 4:** Main results of the included studies analyzing the association between IF, anthropometric data and physical health.

Author, Year	Mean Age (SD)	Significant Physical/Anthropometric Variables	Main Results		Diet Assessment	Adjustment Analysis	Overall Results
			Intervention (If Exists)	Control (If Exists)			
Couto et al., 2025 [26]	72.24 ± 5.15 years	BMI, WC, HC, WHR, SBP, and GGT	- BMI: Δ = −0.62 kg/m^2^ - WC: Δ = −4.5 cm - HC: Δ = −2.0 cm - WHR: Δ = −0.02 - SBP: Δ = −9.8 mmHg	- SBP: Δ = −13.3 mmHg	MEDAS	Age, height, sex, physical activity, hypertension, DM, dyslipidemia, medications, and initial weight.	TRE, combined with a Mediterranean diet, improved some of the anthropometric and biochemical parameters studied compared to the diet alone. Both groups improved in Mediterranean diet adherence, quality of life, and bowel regularity.
			Between-group comparison: - WC: in favor of TRE + MED (*p* = 0.001) - WHR: in favor of TRE + MED (*p* = 0.036) - GGT: in favor of MED-DIET (*p* = 0.020)				
Domaszewski et al., 2020 [27]	IG: 65 ± 4.0 years; CG: 66 ± 4.7 years	Weight, BMI, AFM, and RFM	- Weight: Δ = −1.36 kg - BMI: Δ = −1.29 kg/m^2^ - AFM: Δ = −1.66 kg - RFM: Δ = −1.58%	No significant results	Weekly detailed analysis of the diet consumed	No adjustment	Time-restricted feeding (16:8) over 6 weeks reduced weight, BMI, and body fat in women over 60, without loss of muscle mass and with high adherence.
Kapogiannis et al., 2024 [29]	IG: 63.5 ± 4.2 years CG: 63.0 ± 6.1 years	BMI and WC	- BMI: Δ = −1.41 ± 0.26 kg/m^2^ - WC: Δ = −2.84 ± 0.63 cm	- BMI: Δ = −0.80 ± 0.20 kg/m^2^ - WC: Δ = −1.64 ± 0.82 cm	HL dietary guidelines	Time, group, and time × group interaction	Both 8-week IF and HL diets led to significant reductions in BMI, waist circumference, neuronal insulin resistance and sedentary behavior.
Domaszewski et al., 2022 [28]	IG: 69.3 ± 2.5 years; CG: 69.6 ± 3.3 years	Weight, BMI, AFM, RFM, VFM, and WC	- Weight: Δ = −1.50 kg - BMI: Δ = −0.50 kg/m^2^ - AFM: Δ = −1.00 kg - RFM: Δ = −0.90% - VFM: Δ = −0.55 L - WC: Δ = −2.8 cm	No significant results	Weekly detailed dietary analysis by a professional dietitian	ANCOVA with baseline as covariate	Six-week 16:8 TRE in overweight men led to significant reductions in body weight, BMI, fat mass, WC and VFM, with no loss of muscle mass and a high adherence.
			Between-group comparison: Weight: *p* ≤ 0.001; BMI: *p* ≤ 0.001; AFM: *p* ≤ 0.001; RFM: *p* ≤ 0.003; VFM: *p* ≤ 0.001; WC: *p* ≤ 0.001				
Hussin et al., 2013 [34]	IG: 59.7 ± 6.6 years; CG: 59.7 ± 6.2 years	Weight, BMI, and % body fat	- Weight: Δ = −2.80 kg - BMI: Δ = −1.00 kg/m^2^ - % BF: Δ = −1.5%	No significant results	Diet History Questionnaire (DHQ), 3-day diet record	Age. Reduction in energy intake adjusted for age and body weight	Three months of CR plus IF reduced body weight, BMI, and body fat percentage.
Teng et al., 2013 [33]	IG: 59.6 ± 5.4 years; CG: 59.1 ± 6.2 years	Weight, BMI, % body fat, and FM	- Weight: −2.5 kg - BMI: −0.9 kg/m^2^ - % BF: −1.3% - FM: −1.5 kg - SBP: −6.5 mmHg - DBP: −2.2 mmHg - Total cholesterol: −0.47 mmol/L - LDL-c: −0.33 mmol/L - TC/HDL ratio: −0.22 mmol/L - Total DNA rejoining cells: increased in IG >12 wks (*p* < 0.001) - Total DNA damage: decreased in IG at 6/12 wks - Malondialdehyde (indicator of lipid peroxidation): decreased in IG > 12 wks (*p* < 0.01)	- Total DNA damage score: >12 wks (*p* < 0.05)	7-day food diaries, fasting logs.	Health status and smoking status	Three months of CR plus IF led to significant reductions in weight, BMI, fat percentage, and fat mass. The intervention also improved blood pressure, cholesterol, markers of oxidative stress and DNA damage.
Martens et al., 2020 [31]	64.2 ± 6.1 years	6 min walk distance, heart rate during light exercise and during moderate exercise, glucose tolerance, total cholesterol, LDL-c	Crossover design (No fasting/TRE) - 6 min WD: Δ = +19 m during TRE period - HR LE: Δ = −3 bpm during TRE period - HR ME: Δ = −3 bpm during TRE period - GT: Δ = −1146 mg·min/dL during TRE period - TC: Δ = +11 mg/dL during TRE period - LDL-c: Δ = +11 mg/dL during TRE period		ASA24, Healthy Eating Index (HEI)	Linear mixed models with period and sequence as fixed effects	TRE did not affect body weight, composition, or cognitive function, but modestly improved endurance capacity and GT. TC and LDL-c increased slightly, but no adverse effects were observed.
Tavakoli et al., 2025 [32]	IG: 55.9 ± 8.1 years CG: 58.2 ± 5.1 years	Malondialdehyde (MDA), catalase, Neutrophil-to-Lymphocyte Ratio (NLR), AST, ALT.	- MDA: Δ = −0.01 ± 0.01 µM - Catalase: Δ = +0.02 ± 0.03 µM - NLR: Δ = −0.11 ± 0.21 - AST: Δ = −4.82 ± 0.24 U/L - ALT: Δ = −2.34 ± 0.29 U/L	No significant results	3-day dietary recalls/month, 24 h food records, regular compliance checks	Baseline values. Intention-to-treat analysis used.	8-week 16:8 TRE in postmenopausal, overweight/obese women with rheumatoid arthritis significantly improved oxidative stress, reduced inflammation and lowered liver enzymes compared to controls.
Manoogian et al., 2024 [30]	IG: 56.6 ± 11.5 years CG: 60.6 ± 10.3 years	Weight, BMI, % body fat, % trunk fat, TFM, DBP, and HbA1c	- Weight: Δ = −2.98 kg - BMI: Δ = −1.11 kg/m^2^ - % BF: Δ = −1.36% - % TF: Δ = −1.55% - TFM: Δ = −2240.22 g - DBP: Δ = −3.98 mmHg - HbA1c: Δ = −0.12%	- Weight: Δ = −1.32 kg - BMI: Δ = −0.39	ASA24, myCircadianClock app for tracking dietary timing	Age	3-month 8–10 h TRE in adults with metabolic syndrome led to significant reductions in HbA1c, weight, BMI, body fat, trunk fat, and diastolic BP compared to standard care.
Saini et al., 2022 [40]	≥65 years (mean not explicitly stated)	Weight, circulatory miRNA expression	- Weight: Δ = −2.6 kg - Alteration of 14 miRNA expressions: *p* < 0.05	No control group	No assessment; self-selected fasting/eating windows	No adjustment	4 wk 16:8 TRE in overweight older adults led to significant weight loss and altered the expression of 14 circulatory miRNAs. Downregulated miRNAs target genes involved in cell growth and metabolic pathways, suggesting TRE may promote healthy aging via epigenetic modulation.
Zhao et al., 2022 [41]	63 ± 4 years	Weight, BMI, WC, TFM, VFM, % FM, fasting glucose, HbA1c, and glucose-dependent insulinotropic peptide	- Weight: Δ = −2.3 kg - BMI: Δ = −0.7 kg/m^2^ - WC: Δ = −4 cm - TFM: Δ = −2.1 kg - VFM: Δ = −0.3 kg - % FM: Δ = −1.4% - FG: Δ = −0.3 mmol/L - HbA1c: Δ = −0.2% - GIP premeal at dinner: ↑ after TRE (*p* = 0.003) - GIP AUC postmeal: ↑ with TRE (*p* = 0.007) - Mealtime × TRE on GIP: *p* = 0.009	No control group	myCircadianClock app (photo-based food logging), researcher-estimated daily energy and macronutrient intake from images and annotations	Weight loss	8 wk 14:10 in older men with obesity led to significant reductions in body weight, fat mass, waist circumference, fasting glucose, and HbA1c. TRE improved glycemic control and altered adipose tissue transcriptome, suggesting metabolic benefits independent of major dietary restriction
Mrad et al., 2019 [42]	71 ± 6 years	None	No significant results	No control group	No assessment	No adjustment	Ramadan intermittent fasting did not produce any significant changes in oxidant or antioxidant stress biomarkers, nor in clinical status, in male COPD patients.
Boujelbane et al., 2025 [43]	62.9 ± 4.0 years	Handgrip strength	Active group during Ramadan: No significant changes	Sedentary group during Ramadan: HGS: Nondominant, Δ = −1.18 ± 1.85 kg; Dominant, Δ = −1.05 kg [−0.42; −3.6]	No assessment	Group × Ramadan interaction	During Ramadan intermittent fasting, sedentary participants experienced a decline in muscle strength.
Ezzati et al., 2025 [44]	77.1 ± 6.1 years	None	No significant results	No control group	Fasting/feeding times self-recorded by participants	No adjustment	4 wk 16:8 TRE in overweight older adults showed modest, non-significant reductions in TNF-α and IL-1β, with no change in IL-6, hs-CRP, or 8-isoprostane. TRE was safe, feasible, and showed high adherence.
Anton et al., 2019 [45]	77.1 ± 6.1 years	Weight and BMI	- Weight: Δ = −2.15 ± 1.43 kg - BMI: Δ = −0.9 ± 0.6 kg/m^2^	No control group	Daily diaries	No adjustment	4 wk 16:8 TRE in overweight, sedentary older adults resulted in significant weight and BMI loss. Adherence was high, with minimal adverse events.
Wilkinson et al., 2020 [38]	59 ± 11.1 years	Weight, BMI, % BF, WC, visceral fat rating, SBP, DBP, TC, LDL-c, non-HDL-c, and subjective sleep quality	- Weight: Δ = −3.30 ± 3.20 kg - BMI: Δ = −1.09 ± 0.97 - % BF: Δ = −1.01 ± 0.91 - WC: Δ = −4.46 ± 6.72 cm - Visceral fat rating: Δ = −0.58 ± 0.77 - SBP: Δ = −5.12 ± 9.51 mmHg - DBP: Δ = −6.47 ± 7.94 mmHg - TC: Δ = −13.16 ± 24.29 mg/dL - LDL-c: Δ = −11.94 ± 19.01 mg/dL - Non-HDL-c: Δ = −11.63 ± 22.94 mg/dL - % Days with restful sleep: Δ = +18.3 ± 23.1%	No control group	Participants logged timing and description of all caloric intake through myCircadianClock app	Weight loss and WC	12 wk, 10 h TRE in metabolic syndrome patients significantly reduced weight, body and abdominal fat, blood pressure, cholesterol, and improved subjective sleep quality.
Laatar et al., Nov 2016 [39]	Fallers: 75.43 ± 5.26 years Non-fallers: 72.3 ± 6.42 years	% Center-of-pressure amplitudes (medial–lateral [CoPX] and antero–posterior [CoPY]) with eyes open and closed	CoPX % EO Firm: fallers 43.2% ± 8.6; non-fallers 33% ± 4.5 EC Foam: fallers 61.4% ± 11.4; non-fallers 47.7% ± 9.2 CoPY % EO Firm: fallers 34.1% ± 7.2; non-fallers 20.8% ± 3.9 EC Firm: fallers 37.3% ± 4.4; non-fallers 18.5% ± 3.0 EC Foam: fallers 46.95% ± 9.1; non-fallers 35% ± 6.2 These results were significant between groups No control group		Self-report sleep and eating schedule questionnaire	No adjustment	Ramadan fasting significantly impaired postural control in elderly fallers and non-fallers, with fallers exhibiting greater impairments. Effects were most pronounced in challenging sensory conditions (foam surface) and persisted >3 weeks after Ramadan.
James et al., 2024 [36]	69.7 years	None	No significant results	No control group	REAP-S	No adjustment	8 wk 14:10 nightly fasting in older adults with self-reported memory decline led to no changes in BMI.
Laatar et al., May 2016 [37]	73.33 ± 5.24 years	Center of pressure mean velocity, medio–lateral length and antero–posterior length	Exact numerical results were not reported in the study	No control group	Self-report sleep and eating schedule questionnaire	No adjustment	Ramadan fasting significantly worsened postural balance in elderly men, particularly during the second week. Postural instability persisted under challenging conditions, with partial recovery three weeks post-Ramadan.
Li et al., 2023 [52]	73.38 ± 6.16 years	None	Cross-sectional study No significant results		FFQ	Age, sex, BMI, IADL, illiteracy, widowhood, living alone, occupation, smoking, drinking, night-time feeding, IPAQ, type-2 DM, hypertension, dyslipidemia, stroke, heart disease, and cancer	No significant differences in anthropometrics were found among older adults practicing 14:10 TRE.
Chen et al., 2024 [55]	71.78 ± 5.75 years	Brachial–ankle pulse wave velocity (baPWV), arterial stiffness	Cross-sectional study - baPWV: TRE vs. non-TRE: 1889.28 ± 439.54 vs. 1828.16 ± 405.64 - Adjusted OR for AS Total sample: + Borderline AS: OR = 1.419 (95% CI: 1.077–1.869) + Elevated AS: OR = 1.699 (95% CI: 1.276–2.263) Well-nourished: + Elevated AS: OR = 1.530 (1.107–2.115) At risk of malnutrition: + Borderline AS: OR = 2.270 (1.229–4.190) + Elevated AS: OR = 2.459 (1.287–4.700)		Interview, MNA	Age, sex, BMI, literacy, smoking, alcohol, physical activity, sleep, diabetes, hypertension, and hyperlipidemia	Having a TRE pattern (≤11 h eating window) was associated with higher odds of both borderline and elevated arterial stiffness, independent of many confounders. The association was especially strong in those at risk of malnutrition. In well-nourished individuals, TRE was a significant risk factor only for elevated (not borderline) arterial stiffness.
Estrada-deLeón et al., 2021 [54]	70.96 ± 3.91 years	Lower-extremity function, balance and difficulty to rise from a chair (both from SPPB)	Cross-sectional study - Impaired LEF: + 10–11 h TRE: OR 2.27 (95% CI: 1.56–3.33) + ≥12 h TRE: OR 2.70 (95% CI: 1.80–4.04) - Balance impairment (SPPB): + ≥12 h vs. ≤9 h: OR 2.48 (95% CI: 1.51–4.08) - Difficulty rising from a chair: + ≥12 h vs. ≤9 h: OR 1.47 (95% CI: 1.05–2.06)		MEDAS, dietary recall for habitual food intake and meal timing	Sex, age, energy intake, educational level, smoking, sedentary time, alcohol use, BMI, morbidity, sleep duration, protein intake, Mediterranean diet adherence, and physical activity	Longer nightly fasting periods (≥12 h) in adults ≥64 years were associated with greater odds of impaired physical function, including poorer balance and poorer ability to rise from a chair. The risk of impairment was especially pronounced among those with low physical activity.
Estrada-deLeón et al., 2022 [53]	70.76 ± 3.85 years	HDL-c, potassium, and chloride	Cross-sectional study - HDL-c (mg/dL) Difference for ≥12 h vs. <10 h: −2.79 (95% CI: −4.62, −0.97), *p* = 0.01 - Potassium (mEq/L) Difference for ≥12 h vs. <10 h: +0.11 (95% CI: 0.03–0.19), *p* = 0.01 - Chloride (mEq/L) Difference for ≥12 h vs. <10 h: −0.51 (95% CI: −0.92–0.10), *p* = 0.02		Diet recall with timing of all food occasions, MEDAS	Age, sex, education, smoking, sedentary behavior, physical activity, sleep, alcohol, energy intake, diet quality, MEDAS score, type 2 diabetes, hypertension, hypercholesterolemia, and BMI	Prolonged nightly fasting (≥12 h) was associated with a modest but statistically significant decrease in HDL cholesterol and chloride, and an increase in potassium. This suggests that extended nightly fasting is not beneficial for cardiometabolic health.
Kang et al., 2024 [50]	≥65 years	Hyperglycemia, BMI, LDL-c, TC, triglycerides, Korean Health Eating Index (skipping breakfast, dinner, and no skipping)	Cross-sectional study - Prevalence of hyperglycemia The lowest prevalence of both prediabetes and diabetes was observed in the SD group (*p* < 0.0001) - Risk of hyperglycemia aOR (SD vs. NS) = 0.49 (95% CI: 0.29–0.82) - BMI Significantly higher in SD vs. NS and SB groups - LDL-c SB significantly higher than NS group - TC SB significantly higher than NS group - Triglycerides SB significantly higher than NS group - KHEI SD significantly higher than NS and SB		KHEI, 24 h recall, and FFQ	Sex, energy intake, marital status, income, education, exercise, residential area, smoking, drinking, BMI, and family history	Among elderly Koreans, skipping dinner is associated with a lower risk of hyperglycemia and higher diet quality, while skipping breakfast neither increases nor decreases hyperglycemia risk but is related to poorer dietary patterns and higher atherogenic lipids.
Ooi et al., 2020 [48]	68.53 ± 5.08 years	Weight, BMI, WC, HC, SBP, DBP, fasting glucose, HDL, TG, TC, insulin, malondialdehyde, superoxide dismutase, CRP, and DNA damage	Cohort study - Weight: Δ = −3.65 kg - BMI: Δ = −1.53 kg/m^2^ - WC: Δ = −3.57 cm - HC: Δ = 1.65 cm - SBP: Δ = −7.04 mmHg - DBP: Δ = +0.22 mmHg - Fasting glucose: Δ = −0.37 mmol/L - HDL: Δ = +0.15 mmol/L - TG: Δ = −0.49 mmol/L - TC: Δ = −0.31 mmol/L - Insulin: Δ = −16.25 pmol/L - MDA: Δ = 31.48 nmol/mg - SOD: Δ = −18.38 u.e/min/mg - CRP: Δ = 1.03 nmol/mg - DNA damage: Δ = 3.62% i-IF shows isolated improvements but also worsens several metabolic markers. n-IF exhibits a general decline in metabolic parameters.		No quantitative intake recorded. IF status assigned by fasting regularity (Sunnah fasting: Mon/Thu, sunrise to sunset, and water-only).	Age and education	Over 3 years, older adults who practiced regular intermittent fasting showed significant reductions in weight, BP, glucose, insulin, oxidative stress and DNA damage and increases in HDL and antioxidant enzyme activity. All health improvements were absent in the non-IF group, which showed significant biomarker decline.
Ooi et al., 2022 [49]	68.53 ± 5.08 years	SOD, CRP, insulin, % DNA in tail, and HDL	Cohort study - SOD and CRP: They were full mediators of the effect of regular IF vs. irregular IF on cognitive function (β SOD = 10.04; β CRP = −3.59). - Insulin: Full mediation in regular IF vs. no IF, with significant effects (β = −1.06). - % DNA in tail: Partial mediation in regular IF vs. no IF (β = −1.94). - HDL: Partial mediation in regular IF vs. no IF (β = 4.86).		No quantitative intake recorded. IF status assigned by fasting regularity (Sunnah fasting: Mon/Thu, sunrise to sunset, and water-only).	Age, education, BMI, smoking, hypertension, hypercholesterolemia, and diabetes	Regular IF over 36 months in older adults with mild cognitive impairment led to significant improvements in cognitive function and key metabolic, oxidative stress, inflammation and DNA damage biomarkers. The benefits were mediated mainly by higher SOD (antioxidant), lower CRP (inflammation), lower insulin, lower DNA damage and higher HDL.
Zhang et al., 2024a [56]	69.89 years	CVD mortality, cancer mortality	Cohort study - CVD mortality: >12.38 h fasting had a HR = 1.58 (1.10, 2.28). Per 1 h Increment in Fasting Duration: HR = 1.04 (1.00, 1.09) - Cancer mortality: Per 1 h Increment in Fasting Duration: HR = 0.95 (0.91, 0.98)		Two 24 h dietary recalls	Age, gender, education, marital status, income/poverty, ethnicity, diabetes, CKD, BMI, depression, smoking, alcohol, dietary inflammatory index, hypertension, CVD, shift work, first meal, late eating, sleep, reporter status, and breakfast skipping	Prolonged night-time fasting (>12.4 h) was associated with a higher risk of cardiovascular death compared to intermediate fasting (~11.5 h), which was the safest range. Total and cardiovascular mortality curves showed a U-shaped relationship: both very short and very long fasts increased the risk. This pattern was independent of health status, lifestyle, and diet.

IF: Intermittent fasting; BMI: body mass index; WC: waist circumference; HC: hip circumference; WHR: waist-to-hip ratio; SBP: systolic blood pressure; GGT: gamma-glutamyl transferase; MEDAS: Mediterranean Diet Adherence Screener; IG: intervention group; CG: control group; AFM: absolute fat mass; RFM: relative fat mass; HL: healthy living (diet); VFM: visceral fat mass; CR: caloric restriction; % BF: percent body fat; FM: fat mass; DBP: diastolic blood pressure; LDL-c: low-density lipoprotein cholesterol; TC: total cholesterol; HDL-c: high-density lipoprotein cholesterol; DNA: deoxyribonucleic acid; 6 min WD: six-minute walk distance; HR LE: heart rate during light exercise; HR ME: heart rate during moderate exercise; GT: glucose tolerance; ASA24: automated self-administered 24 h dietary assessment; HEI: Healthy Eating Index; MDA: malondialdehyde; NLR: neutrophil-to-lymphocyte ratio; AST: aspartate aminotransferase; ALT: alanine aminotransferase; TFM: trunk fat mass; FG: fasting glucose; GIP: glucose-dependent insulinotropic peptide; AUC: area under the curve; miRNA: microRNA; COPD: chronic obstructive pulmonary disease; HGS: handgrip strength; TNF-α: tumor necrosis factor alpha; IL-1β: interleukin-1 beta; IL-6: interleukin-6; hs-CRP: high-sensitivity C-reactive protein; SPPB: Short Physical Performance Battery; LEF: lower-extremity function; EO: eyes open; EC: eyes closed; CoPX: center-of-pressure medial–lateral amplitude; CoPY: center-of-pressure antero–posterior amplitude; FFQ: Food Frequency Questionnaire; IADL: Instrumental Activities of Daily Living; IPAQ: International Physical Activity Questionnaire; MNA: Mini Nutritional Assessment; AS: arterial stiffness; HDL: high-density lipoprotein; TG: triglycerides; CRP: C-reactive protein; i-IF: intermittent fasting group (regular IF); n-IF: non-intermittent fasting group (no or irregular IF); CVD: cardiovascular disease; HR: hazard ratio; CKD: chronic kidney disease; ↑: increased.

**Table 5:** Main results of the included studies analyzing the association between IF and mental health.

Author, Year	Age Mean (SD)	Significant Mental Health Variables	Main Results		Mental Health Assessment	Adjustment Analysis	Overall Results
			Intervention (If Exists)	Control (If Exists)			
Hussin et al., 2013 [34]	Int: 59.7 ± 6.6 years; Ctrl: 59.7 ± 6.2 years	Tension, anger, confusion, vigor, and total mood disturbance	- Tension: Δ = −1.0 - Anger: Δ = −1.9 - Confusion: Δ = −2.0 - Vigor: Δ = +1.6 - TMD: Δ = −10.4	No significant results	Profile of Mood States (POMS)	Age	Three months of CR plus IF reduced negative mood states (tension, anger, confusion, and total mood disturbance) and increased vigor. No significant changes in depression scores.
Boujelbane et al., 2025 [43]	62.9 ± 4.0 years	Anxiety (GAD-7), depression (GDS)	Active group during Ramadan: GAD-7: Δ = −3 [−2.25; −12] GDS: Δ = −1 [0; −4]	Sedentary group during Ramadan: GAD-7: Δ = −3 [0; −10] GDS: Δ = −1 [0; −3]	GAD-7 score, GDS score	Group × Ramadan interaction	During Ramadan IF, anxiety and depression improved significantly in older adults.
Boujelbane et al., 2022 [35]	62.93 ± 3.99 years	None	No significant results	No control group	GAD-7 score, GDS score	Group × Ramadan interaction	No significant changes in mental health variables were reported. Sleep quality and insomnia worsened in both groups, more so in sedentary participants.
James et al., 2024 [36]	69.7 years	Insomnia	ISI: Δ = −1.72	No control group	Insomnia Severity Index	No adjustment	Eight weeks of 14 h nightly fasting in older adults with self-reported memory decline significantly reduced insomnia severity.
Lee et al., 2020 [46]	77.1 years	None	No significant results	No control group	Diet satisfaction survey	No adjustment	No significant changes in mood. Most participants found time-restricted eating simple and were motivated by weight loss. Understanding varied, highlighting the need for better education and support.
Currenti et al., Jun 2021 [51]	>70 years	Mental distress	Cross-sectional study Mental distress: OR = 0.14 (95% CI: 0.03–0.65)		PSQI, PSS, CES-D-10. FFQ for dietary assessment	Age, sex, total energy intake, education, occupation, smoking, physical activity, health status, Mediterranean diet adherence, and having breakfast/dinner	Restricting the daily feeding window to 8 h or less (TRF) among elderly Italian adults (>70 y) was associated with a substantially lower likelihood of signs of mental health distress, independent of Mediterranean diet quality and dinner habits.
Li et al., 2023 [52]	73.38 ± 6.16 years	None	Cross-sectional study No significant results		GDS, sleep duration	Age, sex, BMI, IADL, illiteracy, widowhood, living alone, occupation, smoking, drinking, night-time feeding, IPAQ, type-2 DM, hypertension, dyslipidemia, stroke, heart disease, and cancer	No significant differences in depression or sleep duration were found among older adults practicing 14:10 TRE.
Estrada-deLeón et al., 2021 [54]	70.96 ± 3.91 years	Depression	Cross-sectional study Significant higher prevalence of depression in those with ≥12 h fasting (*p* = 0.001)		Diagnosis	Sex, age, energy intake, educational level, smoking, sedentary time, alcohol use, BMI, morbidity, sleep duration, protein intake, Mediterranean diet adherence, and physical activity	Higher prevalence of depression in those with ≥12 h fasting, but the main results remained after excluding depressed participants.

Int: Intervention; Ctrl: control; TMD: total mood disturbance; POMS: Profile of Mood States; CR: caloric restriction; IF: intermittent fasting; GAD-7: Generalized Anxiety Disorder-7; GDS: Geriatric Depression Scale; ISI: Insomnia Severity Index; OR: odds ratio; CI: confidence interval; PSQI: Pittsburgh Sleep Quality Index; PSS: Perceived Stress Scale; CES-D-10: 10-item Center for Epidemiologic Studies Depression Scale; FFQ: Food Frequency Questionnaire; TRF: time-restricted feeding; BMI: body mass index; IADL: Instrumental Activities of Daily Living; IPAQ: International Physical Activity Questionnaire; DM: diabetes mellitus; TRE: time-restricted eating.

**Table 6:** Main results of the included studies analyzing the association between IF and cognitive health.

Author, Year	Age Mean (SD)	Significant Cognitive Variables	Main Results		Cognitive Assessment	Adjustment Analysis	Overall Results
			Intervention (If Exists)	Control (If Exists)			
Kapogiannis et al., 2024 [29]	IF: 63.5 ± 4.2 years Healthy Living Diet: 63.0 ± 6.1 years	Executive function composite (EFC), fluency factor (FF), dimensional set shifting (DSS), short-delay cued recall (SDCR), long-delay cued recall (LDCR), and brain-age estimates (BAE)	- EFC: Improved - FF: Improved - DSS: Improved - SDCR: Improved - LDCR: Improved - BAE: 2.63-year decrease	- EFC: Improved - BAE: 2.42-year decrease	NIH Examiner, California Verbal Learning Test (CVLT)	Time, group, and time/group	IF improved all cognitive domains and reduced brain age by 2.63 years.
Boujelbane et al., 2022 [35]	Active group: 63.19 ± 4.56 years Sedentary group: 62.71 ± 3.52 years	Executive function, attention, inhibition, associative memory, recognition memory, and associative learning	Active group during Ramadan: - Executive function: ↑ (*p* = 0.035) - Attention: ↑ (*p* = 0.005) - Inhibition: ↑ (*p* = 0.02) - Associative memory: ↑ (*p* = 0.041) - Recognition memory: ↑ (*p* = 0.025)	Sedentary group during Ramadan: - Associative learning: ↓ (*p* = 0.041)	Neurotrack digital cognitive battery	Group × Ramadan interaction	4 wk Ramadan IF improved executive function, attention, inhibition, associative and recognition memory in physically active older adults, while sedentary participants experienced a decline in associative learning. Both groups had worsened sleep quality.
Boujelbane et al., 2025 [43]	62.9 ± 4.0 years	Vigilance performance	Active group during Ramadan: Vigilance reaction time: Δ = −94.5 [−63.5; −344]	Sedentary group during Ramadan: Vigilance reaction time: −64 [20.75; −315]	Psychomotor vigilance test reaction time	Group × Ramadan interaction	Ramadan intermittent fasting improved vigilance significantly in older adults—especially those who were physically active.
Anton et al., 2019 [45]	77.1 ± 6.1 years	None	No significant results	No control group	MoCA score	No adjustment	4 wk 16:8 TRE in overweight, sedentary older adults led to no significant changes in cognitive or physical function.
Laatar et al., Nov 2016 [39]	Fallers: 75.43 ± 5.26 years Non-fallers: 72.3 ± 6.42 years	Simple reaction time (second and fourth week of Ramadan compared to baseline)	SRT fallers - SWR: Δ = +99 ms - FWR: Δ = +65 ms SRT non-fallers - SWR: Δ = +98 ms - FWR: Δ = +78 ms	No control group	SRT test	No adjustment	Ramadan significantly increased (worse) reaction time during the second week and the fourth week of Ramadan compared to baseline (before Ramadan) in both groups.
Martens et al., 2020 [31]	64.2 ± 6.1 years	None	No significant results	Crossover design	NIH Toolbox Cognition Battery	Period, sequence	All participants had above-average cognitive function at baseline, and no improvement was observed after intervention.
Domaszewski et al., 2022 [28]	IG: 69.3 ± 2.5 years; CG: 69.6 ± 3.3 years	None	No significant results	No significant results	MMSE	Baseline	All participants had MMSE >23 (no cognitive impairment), and no significant change was observed.
James et al., 2024 [36]	69.7 years	Cognitive function	MAPS: Δ = +11.88	No control group	Memory and Attention Phone Screener	No adjustment	Eight weeks of 14 h nightly fasting in older adults with self-reported memory decline significantly improved global cognitive function.
Laatar et al., May 2016 [37]	73.33 ± 5.24 years	Simple reaction time (second and fourth week of Ramadan compared to baseline)	- SWR: Δ = +94.57 ms - FWR: Δ = +64.47 ms	No control group	SRT test	No adjustment	Ramadan significantly increased (worse) reaction time during the second week and the fourth week of Ramadan compared to baseline (before Ramadan) in both groups.
Lee et al., 2020 [46]	77.1 years	None	No significant results	No control group	Diet satisfaction survey	No adjustment	No significant changes in cognitive function.
Li et al., 2023 [52]	73.38 ± 6.16 years	MMSE, orientation to place, and attention/calculation	Cross-sectional study - MMSE: Lower in TRF group (22.45 ± 4.63) vs. no restriction (24.97 ± 4.24), *p* < 0.001. - OtP: Lower in TRF (3.65 ± 0.83) vs. no restriction (4.73 ± 0.71), *p* < 0.001. - A/C: Lower in TRF (2.36 ± 1.64) vs. no restriction (3.64 ± 1.60), *p* < 0.001.		MMSE	Age, sex, BMI, IADL, illiteracy, widowhood, living alone, occupation, smoking, drinking, night-time feeding, IPAQ, type-2 DM, hypertension, dyslipidemia, stroke, heart disease, and cancer	Consuming all daily meals within a ≤10 h eating window (TRF) is associated with a significantly higher prevalence of cognitive impairment and particularly lower scores in “orientation to place” and “attention/calculation” domains of the MMSE.
Currenti et al., Jan 2021 [47]	65.1 ± 9.6 years	Cognitive impairment	Cross-sectional study - TRE vs. No fasting Adjusted OR = 0.28 (95% CI: 0.07–0.90) - Consuming breakfast vs. no consumption Adjusted OR = 0.37 (95% CI: 0.16–0.89)		SPMSQ, FFQ for meal assessment	Age, sex, marital status, education level, occupational status, smoking, alcohol, physical activity, BMI, diabetes, hypertension, dyslipidemia, CVD, and cancer	Individuals practicing 14:10 TRE were significantly less likely to have cognitive impairment than those with longer eating windows. The effect was strongest when TRE included breakfast, suggesting alignment with circadian rhythms may be key.
Ooi et al., 2020 [48]	68.53 ± 5.08 years	Cognitive function	Cohort study - MMSE: 6.43 - MoCA: 5.16 - RAVLT: 6.05 - Digit Span Test: 1.04 - Digit Symbol: 1.28		MMSE, MoCA, RAVLT, Digit Span Test, and Digit Symbol	Age and education	Over 3 years, older adults with MCI who practiced regular intermittent fasting showed significant improvement in global cognitive function and all cognitive domains. All health improvements were absent in the non-IF group, which showed significant cognitive decline.
Ooi et al., 2022 [49]	68.53 ± 5.08 years	Cognitive function	Cohort study - MMSE: Regular fasting = 24.05 ± 3.25 vs. no fasting = 16.33 ± 4.11 (*p* < 0.05) - MoCA: Regular fasting = 19.43 ± 4.45 vs. no fasting = 14.37 ± 4.25 (*p* < 0.05) - Digit Span Test: Regular fasting = 8.88 ± 2.38 vs. no fasting = 5.73 ± 2.44 (*p* < 0.05) - RAVLT: Regular fasting = 36.05 ± 0.61 vs. no fasting = 20.41 ± 0.71 (*p* < 0.05)		MMSE, MoCA, digit span test, and RAVLT	Age, education, BMI, smoking, hypertension, hypercholesterolemia, and diabetes	Regular IF over 36 months in older adults with mild cognitive impairment led to significant improvements in cognitive function.

IF: Intermittent fasting; EFC: executive function composite; FF: fluency factor; DSS: dimensional set shifting; SDCR: short-delay cued recall; LDCR: long-delay cued recall; BAE: brain-age estimates; NIH: National Institutes of Health; CVLT: California Verbal Learning Test; TRF: the Time-restricted feeding; MAPS: Memory and Attention Phone Screener; SRT: simple reaction time; SWR: second week of Ramadan; FWR: fourth week of Ramadan; IG: intervention group; CG: control group; MMSE: Mini-Mental State Examination; TRE: time-restricted eating; OtP: orientation to place; A/C: attention/calculation; OR: odds ratio; CI: confidence interval; SPMSQ: Short Portable Mental Status Questionnaire; FFQ: Food Frequency Questionnaire; BMI: body mass index; IADL: Instrumental Activities of Daily Living; IPAQ: International Physical Activity Questionnaire; DM: diabetes mellitus; CVD: cardiovascular disease; MoCA: Montreal Cognitive Assessment; RAVLT: Rey Auditory Verbal Learning Test; ↑: increased; ↓: decreased

**Table 7:** Estimated effects of dietary interventions compared with the UD on weight loss.

Weight (kg)				
Intervention	MD	95% CI	z	*p*-Value
TRE 16:8	−1.92	−2.57; −1.27	−5.79	**<0.0001**
TRE 12:12	−1.16	−3.95; 1.64	−0.81	0.418
ISF	−2.36	−2.94; −1.79	−8.06	**<0.0001**
IF 5:2	−1.99	−4.40; 0.42	−1.62	0.106
HLD	−0.26	−1.74; 1.23	−0.34	0.734
UD	Ref.	Ref.	Ref.	Ref.

Abbreviations: TRE 16:8: Time-restricted eating with 16 h fasting and 8 h eating window; TRE 12:12: time-restricted eating with 12 h fasting and 12 h eating window; ISF: Islamic sunnah fasting; IF 5:2: IF with 5 days of normal eating and 2 days of caloric restriction; HLD: healthy living diet; UD: usual diet; MD: mean difference; CI: confidence interval. Statistical significance was set at *p* < 0.05, and significant results are highlighted in bold.

**Table 8:** P-score rankings for weight loss and BMI reduction across interventions.

P-Score Ranking Table			
Weight Loss		BMI Loss	
Intervention	P-Score	Intervention	P-Score
ISF	0.850	TRE 16:8	0.751
IF 5:2	0.704	IF 5:2	0.729
TRE 16:8	0.667	ISF	0.618
TRE 12:12	0.471	TRE 12:12	0.499
HLD	0.182	HLD	0.259
UD	0.126	UD	0.143

Abbreviations: TRE 16:8: Time-restricted eating with 16 h fasting and 8 h eating window; TRE 12:12: time-restricted eating with 12 h fasting and 12 h eating window; ISF: Islamic sunnah fasting; IF 5:2: IF with 5 days of normal eating and 2 days of caloric restriction; HLD: healthy living diet; UD: usual diet; BMI: body mass index.

**Table 9:** Estimated effects of dietary interventions compared with the UD on BMI loss.

BMI (kg/m^2^)				
Intervention	MD	95% CI	z	*p*-Value
TRE 16:8	−1.01	−1.69; −0.33	−2.90	**0.004**
TRE 12:12	−0.61	−2.52; 1.30	−0.63	0.530
ISF	−0.81	−1.43; −0.19	−2.55	**0.011**
IF 5:2	−1.04	−2.61; 0.53	−1.30	0.193
HLD	−0.24	−1.44; 0.96	−0.40	0.693
UD	Ref.	Ref.	Ref.	Ref.

Abbreviations: TRE 16:8: Time-restricted eating with 16 h fasting and 8 h eating window; TRE 12:12: time-restricted eating with 12 h fasting and 12 h eating window; ISF: Islamic sunnah fasting; IF 5:2: IF with 5 days of normal eating and 2 days of caloric restriction; HLD: healthy living diet; UD: usual diet; MD: mean difference; CI: confidence interval; BMI: body mass index. Statistical significance was set at *p* < 0.05, and significant results are highlighted in bold.

## Data Availability

All data generated or analyzed during this study are included in this published article and its Supplementary Material.

## Supplementary Materials

The following supporting information can be downloaded at: https://www.mdpi.com/article/10.3390/nu18091450/s1, Supplementary Material S1: Search strategy; Supplementary Material S2: Figure S1: Funnel plot for assessment of publication bias in network meta-analysis of intermittent fasting interventions for weight loss; Figure S2: Funnel plot for assessment of publication bias in network meta-analysis of intermittent fasting interventions for BMI loss; Supplementary Material S3: Table S1: Assessment of risk of bias in randomized trials (RoB 2); Supplementary Material S4: Table S2: Quality assessment of controlled intervention studies; Table S3: Quality assessment tool for observational cohort and cross-sectional studies; Table S4: Quality assessment tool for before–after (pre–post) studies with no control group.

## Abbreviations

IF	Intermittent fasting
TRE	Time-restricted eating
ISF	Islamic Sunnah fasting
NMA	Network meta-analysis
RCTs	Randomized controlled trials
ADF	Alternate-day fasting
BMI	Body mass index
HbA1c	Hemoglobin A1c
LDL	Low-density lipoprotein
MMSE	Mini-Mental State Examination
MoCA	Montreal Cognitive Assessment
RAVLT	Rey Auditory Verbal Learning Test
MAPS	Memory and Aging Performance Scale
NIH	National Institutes of Health
PICOS	Population, Intervention, Comparison, Outcomes, Study design
PRISMA	Preferred Reporting Items for Systematic Reviews and Meta-Analyses
PROSPERO	International Prospective Register of Systematic Reviews
MD	Mean difference
RoB 2.0	Risk of Bias 2.0
ROBINS-I	Risk Of Bias In Non-randomized Studies of Interventions
SUCRA	Surface Under the Cumulative Ranking curve
CINeMA	Confidence In Network Meta-Analysis
CI	Confidence interval
HLD	Healthy living diet
UD	Usual diet
GDS	Geriatric Depression Scale
POMS	Profile of Mood States
GAD-7	Generalized Anxiety Disorder-7
ISI	Insomnia Severity Index
OR	Odds ratio
AST	Aspartate aminotransferase
ALT	Alanine aminotransferase
MDA	Malondialdehyde
SOD	Superoxide dismutase
