# Supplementary material for: Intermittent Fasting and Healthy Aging in Older Adults: A Systematic Review of Cardiometabolic, Mental Health and Cognitive Outcomes with a Network Meta-Analysis of Anthropometric Measures
Source: Nutrients. 2026 Apr 30;18(9):1450. doi: 10.3390/nu18091450 (PMC13165003; doi:10.3390/nu18091450)
Supplement: Supplementary file 1 [file nutrients-18-01450-s001.zip › Supplementary material S1.pdf]

Supplementary material S1. Search strategy

PUBMED

("time-restricted eating"[Title/Abstract] OR "time-restricted feeding"[Title/Abstract] OR "time-restricted fasting"[Title/Abstract] OR "time-restricted diet"[Title/Abstract] OR "feeding window"[Title/Abstract] OR "circadian fasting"[Title/Abstract] OR "chrono-nutrition"[Title/Abstract] OR "intermittent fasting"[Title/Abstract] OR "meal skipping"[Title/Abstract] OR "alternate-day fasting"[Title/Abstract] OR "periodic fasting"[Title/Abstract] OR "intermittent energy restriction"[Title/Abstract]) AND ("older adults"[Title/Abstract] OR "elderly"[Title/Abstract] OR "aged"[Title/Abstract] OR "older people"[Title/Abstract] OR "senior"[Title/Abstract] OR "geriatric"[Title/Abstract]) AND ("physical health"[Title/Abstract] OR "cardiometabolic health"[Title/Abstract] OR "body weight"[Title/Abstract] OR "weight"[Title/Abstract] OR "blood pressure"[Title/Abstract] OR "hypertension"[Title/Abstract] OR "body composition"[Title/Abstract] OR "body mass index"[Title/Abstract] OR "waist circumference"[Title/Abstract] OR "waist-to-hip ratio"[Title/Abstract] OR "abdominal fat"[Title/Abstract] OR "fat mass"[Title/Abstract] OR "metabolic health"[Title/Abstract] OR "glucose metabolism"[Title/Abstract] OR "glucose"[Title/Abstract] OR "insulin"[Title/Abstract] OR "insulin resistance"[Title/Abstract] OR "blood glucose"[Title/Abstract] OR "fasting glucose"[Title/Abstract] OR "glycemic response"[Title/Abstract] OR "cholesterol"[Title/Abstract] OR "triglycerides"[Title/Abstract] OR "low-density lipoprotein cholesterol"[Title/Abstract] OR "high-density lipoprotein cholesterol"[Title/Abstract] OR "inflammation"[Title/Abstract] OR "mental health"[Title/Abstract] OR "mood"[Title/Abstract] OR "depression"[Title/Abstract] OR "stress"[Title/Abstract] OR "anxiety"[Title/Abstract] OR "psychological well-being"[Title/Abstract] OR "mental well-being"[Title/Abstract])

SCIENCEDIRECT

("time-restricted eating" OR "intermittent fasting") AND ("older adults" OR "aged") AND ("health" OR "diseases" OR "cardiovascular" OR "cognitive" OR "metabolic")

SCOPUS

(TITLE-ABS-KEY ("time-restricted eating") OR TITLE-ABS-KEY ("time-restricted feeding") OR TITLE-ABS-KEY ("intermittent fasting") OR TITLE-ABS-KEY ("alternate-day fasting") OR TITLE-ABS-KEY ("intermittent energy restriction") OR TITLE-ABS-KEY ("meal skipping")) AND (TITLE-ABS-KEY ("older adults") OR TITLE-ABS-KEY ("elderly") OR TITLE-ABS-KEY ("aged")) AND (TITLE-ABS-KEY ("health") OR TITLE-ABS-KEY ("cardiovascular") OR TITLE-ABS-KEY ("depression") OR TITLE-ABS-KEY ("anxiety") OR TITLE-ABS-KEY ("mental health") OR TITLE-ABS-KEY ("body composition") OR TITLE-ABS-KEY ("blood") OR TITLE-ABS-KEY ("weight") OR TITLE-ABS-KEY ("metabolic") OR TITLE-ABS-KEY ("inflammation") OR TITLE-ABS-KEY ("glucose") OR TITLE-ABS-KEY ("insulin") OR TITLE-ABS-KEY ("well-being"))

AND (LIMIT-TO (DOCTYPE , "ar")) AND (LIMIT-TO (LANGUAGE , "English") OR LIMIT-TO (LANGUAGE , "Spanish"))
