# Supplementary material for: Intermittent Fasting and Healthy Aging in Older Adults: A Systematic Review of Cardiometabolic, Mental Health and Cognitive Outcomes with a Network Meta-Analysis of Anthropometric Measures
Source: Nutrients. 2026 Apr 30;18(9):1450. doi: 10.3390/nu18091450 (PMC13165003; doi:10.3390/nu18091450)

Supplementary material S2

Fig S1. Funnel plot for assessment of publication bias in network meta-analysis of intermittent fasting interventions for weight loss.

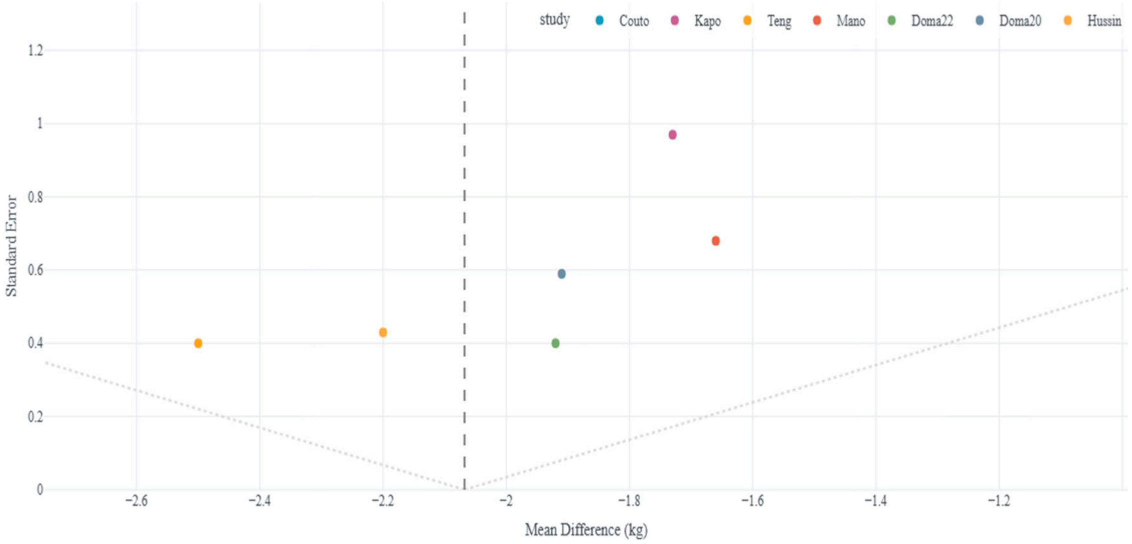

Fig S2. Funnel plot for assessment of publication bias in network meta-analysis of intermittent fasting interventions for BMI loss.

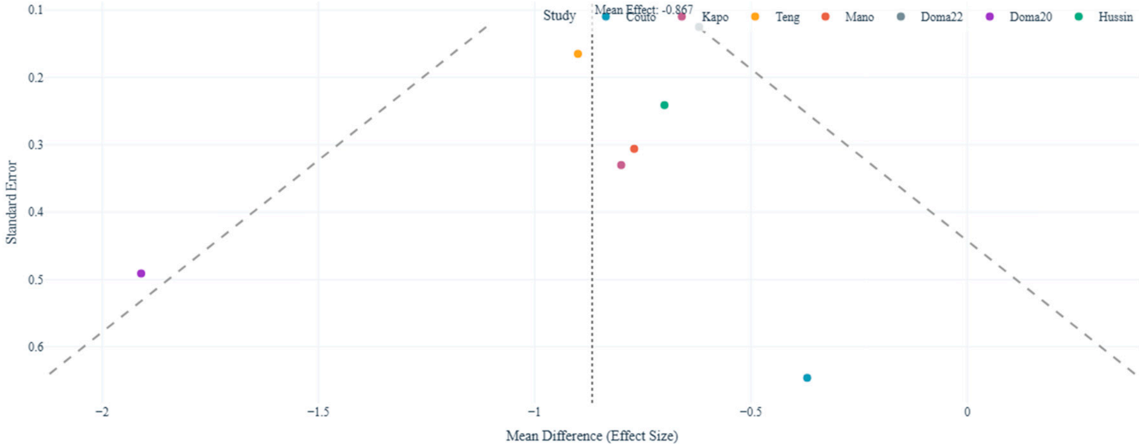

Supplement: Supplementary file 1 [file nutrients-18-01450-s001.zip › Supplementary material S2.pdf]
