# Supplementary material for: Intermittent Fasting and Healthy Aging in Older Adults: A Systematic Review of Cardiometabolic, Mental Health and Cognitive Outcomes with a Network Meta-Analysis of Anthropometric Measures
Source: Nutrients. 2026 Apr 30;18(9):1450. doi: 10.3390/nu18091450 (PMC13165003; doi:10.3390/nu18091450)
Supplement: Supplementary file 1 [file nutrients-18-01450-s001.zip › Supplementary material S3.pdf]

Supplementary material S3

Table S1. *Assessment of Risk of Bias In Randomized Trials (RoB 2)*

Study	Domains of Risk of Bias					Overall Risk
	D1: Risk of bias arising from the randomization process	D2: Risk of bias due to deviations from the intended interventions	D3: Missing outcome data	D4: Risk of bias in measurement of the outcome	D5: Risk of bias in selection of the reported result	
Couto et al., 2025	Low	Low	Low	Low	Low	**Low risk**
Domaszewski et al., 2020	Some concerns (U)	Low	Low	Low	Some concerns (U)	**Some concerns**
Domaszewski et al., 2022	Some concerns (U)	Low	Low	Low	Some concerns (U)	**Some concerns**
Hussin et al., 2013	Some concerns (U)	Low	Low	Low	Some concerns (U)	**Some concerns**
Kapogiannis et al., 2024	Low	Some concerns (TN)	Low	Low	Low	**Some concerns**
Manoogian et al., 2024	Low	Low	Low	Low	Low	**Low risk**
Martens et al., 2020	Low	Low	Low	Low	Low	**Low risk**

Tavakoli et al., 2025	Low	Low	Low	Low	Low	**Low risk**
Teng et al., 2013	Some concerns (U)	Low	Low	Low	Some concerns (U)	**Some concerns**

Direction of Bias: U (unpredictable); TN (towards the null); AN (away from the null).
