# Supplementary material for: Intermittent Fasting and Healthy Aging in Older Adults: A Systematic Review of Cardiometabolic, Mental Health and Cognitive Outcomes with a Network Meta-Analysis of Anthropometric Measures
Source: Nutrients. 2026 Apr 30;18(9):1450. doi: 10.3390/nu18091450 (PMC13165003; doi:10.3390/nu18091450)
Supplement: Supplementary file 1 [file nutrients-18-01450-s001.zip › Supplementary material S4.pdf]

Supplementary material S4

Table S2. Quality Assessment of Controlled Intervention Studies.

Study	1	2	3	4	5	6	7	8	9	10	11	12	13	14	R
Couto et al., 2025	Y	Y	CD	N	NR	Y	Y	Y	Y	Y	Y	N	Y	N	**64.3%**
Domaszewski et al., 2020	Y	CD	NR	N	NR	Y	Y	Y	Y	Y	Y	Y	Y	N	**64.3%**
Domaszewski et al., 2022	Y	Y	CD	N	NR	Y	Y	Y	Y	Y	Y	Y	Y	N	**71.4%**
Hussin et al., 2013	Y	CD	NR	N	NR	Y	Y	Y	Y	Y	Y	Y	Y	N	**64.3%**
Kapogiannis et al., 2024	Y	Y	Y	N	CD	Y	Y	Y	Y	Y	Y	Y	Y	N	**78.6%**
Manoogian et al., 2024	Y	Y	Y	N	NR	Y	Y	Y	Y	Y	Y	Y	Y	Y	**58.7%**
Martens et al., 2020	Y	Y	NR	N	Y	Y	Y	Y	Y	Y	Y	Y	Y	Y	**85.7%**
Tavakoli et al., 2025	Y	CD	NR	N	NR	Y	Y	Y	Y	Y	Y	CD	Y	CD	**57.1%**
Teng et al., 2013	Y	CD	NR	N	NR	Y	Y	Y	Y	Y	Y	CD	Y	CD	**57.1%**

Quality assessment of included studies using the NIH Study Quality Assessment Tool for Controlled Intervention Studies.

Table S3. Quality Assessment Tool for Observational Cohort and Cross-Sectional Studies

Study	1	2	3	4	5	6	7	8	9	10	11	12	13	14	R
Chen et al., 2024	Y	Y	Y	Y	N	N	N	Y	Y	NA	Y	NR	NA	Y	**66.7%**
Currenti et al., Jan 2021	Y	Y	Y	Y	N	N	N	Y	Y	NA	Y	NR	NA	Y	**66.7%**
Currenti et al., Jun 2021	Y	Y	Y	Y	N	N	N	Y	Y	NA	Y	NR	NA	Y	**66.7%**
Estrada-De León et al., 2021	Y	Y	Y	Y	N	N	N	Y	Y	NA	Y	NR	NA	Y	**66.7%**
Estrada-De León et al., 2022	Y	Y	Y	Y	N	N	N	Y	Y	NA	Y	Y	NA	Y	**75%**
Kang et al., 2024	Y	Y	Y	Y	N	N	N	Y	Y	NA	Y	NR	NA	Y	**66.7%**
Li et al., 2023	Y	Y	Y	Y	N	N	N	Y	Y	NA	Y	NR	NA	Y	**66.7%**
Ooi et al., 2020	Y	Y	Y	Y	Y	Y	Y	Y	Y	Y	Y	NR	Y	Y	**92.9%**
Ooi et al., 2022	Y	Y	Y	Y	Y	Y	Y	Y	Y	Y	Y	NR	Y	Y	**92.9%**
Zhang et al., 2024a	Y	Y	Y	Y	NR	Y	Y	Y	Y	Y	Y	Y	Y	Y	**92.9%**

Table S4. Quality Assessment Tool for Before-After (Pre-Post) Studies With No Control Group

Study	1	2	3	4	5	6	7	8	9	10	11	12	R
Anton et al., 2019	Y	Y	Y	CD	N	Y	Y	NR	Y	Y	N	NA	**63.6%**
Boujelbane et al., 2022	Y	Y	Y	CD	Y	Y	Y	NR	Y	Y	N	NA	**72.7%**
Boujelbane et al., 2025	Y	Y	Y	CD	N	Y	Y	NR	Y	Y	N	NA	**63.6%**
Ezzati et al., 2025	Y	Y	Y	CD	N	Y	Y	NR	Y	Y	N	NA	**63.6%**
James et al., 2024	Y	Y	Y	CD	N	Y	Y	NR	Y	Y	N	NA	**63.6%**
Mrad et al., 2019	Y	Y	Y	CD	N	Y	Y	NR	Y	Y	N	NA	**63.6%**
Laatar et al., May 2016	Y	Y	Y	CD	N	Y	Y	NR	Y	Y	N	NA	**63.6%**
Laatar et al., Nov 2016	Y	Y	Y	CD	N	Y	Y	NR	Y	Y	N	NA	**63.6%**
Lee et al., 2020	Y	Y	Y	CD	N	Y	Y	NR	Y	N	N	NA	**54.5%**
Saini et al., 2022	Y	Y	Y	CD	N	Y	Y	NR	Y	Y	N	NA	**63.6%**
Wilkinson et al., 2020	Y	Y	Y	CD	N	Y	Y	NR	Y	Y	N	NA	**63.6%**
Zhao et al., 2022	Y	Y	Y	CD	N	Y	Y	NR	Y	Y	N	NA	**63.6%**
